# Plant-based diets and risk of type 2 diabetes: systematic review and dose–response meta-analysis

**DOI:** 10.1017/S0007114525104157

**Published:** 2025-08-28

**Authors:** Alberto Murciano, Marcella Malavolti, Susan Fairweather-Tait, Stefania Paduano, Marco Vinceti, Tommaso Filippini

**Affiliations:** 1 CREAGEN-Environmental, Genetic and Nutritional Epidemiology Research Center, Department of Biomedical, Metabolic and Neural Sciences, University of Modena and Reggio Emilia, Modena, Italy; 2 Norwich Medical School, University of East Anglia, Norwich, UK; 3 Department of Epidemiology, Boston University School of Public Health, Boston, MA, USA; 4 School of Public Health, University of California Berkeley, Berkeley, CA, USA

**Keywords:** Diabetes, Plant-based diet, Healthy plant-based diet, Processed foods, Vegan diet, Vegetarian diet

## Abstract

Type 2 diabetes (T2D) incidence has been steadily increasing over the past few decades. Several studies have evaluated the effect of plant-based, vegetarian or vegan diets on the risk of T2D, although their potential benefits need to be confirmed and characterised. We performed a literature search up to 10 July 2025, using the terms/keywords related to plant-based index (PDI), vegetarian/vegan diets and T2D. We included observational non-experimental studies evaluating adherence to such diets in adult subjects assessing T2D risk. We specifically considered overall PDI and related healthy PDI (hPDI) and unhealthy PDI (uPDI), assessing intake of different food groups. We included 36 studies published between 1999 and 2025. We found an inverse association between adherence to vegetarian/plant-based dietary patterns and T2D risk. This association was stronger, though statistically imprecise, for the vegan diet (RR = 0·65, 95 % CI 0·42, 1·00) and for lacto-ovo-vegetarian diet (RR = 0·68, 0·57, 0·82). For studies using plant-based indices, the RR were 0·82 (0·69, 0·82), 0·76 (0·69, 0·82) and 1·13 (0·98, 1·30) for overall PDI, hPDI and uPDI, respectively. In the dose–response meta-analysis, overall PDI and hPDI showed an inverse and almost linear association with T2D risk. Conversely, adherence to uPDI directly correlated with T2D risk. Overall, adherence to vegan/vegetarian diets may reduce T2D risk, while an unhealthy plant-based diet appears to linearly increase disease risk, indicating caution in the consumption of such unhealthy foods even if of plant origin. The beneficial association between vegetarian and healthy plant-based diets may have major public health implications.

Highlights

- Intake of healthy foods such as vegetables, legumes and whole grains protects against type 2 diabetes (T2D).
- The association between a healthy plant-based diet and T2D is almost linear.
- Unhealthy plant-based diets are not beneficial and may even increase risk of T2D.
- Limitation of unhealthy plant-based foods, even if they are of plant origin, is recommended.

## Introduction

Type 2 diabetes (T2D) is a chronic metabolic disease with an estimated 537 million affected adults aged 20–79 years worldwide (namely, 10·5 % of all adults in this age group)^(1)^. By 2030, projections show that 643 million, and by 2045, 783 million adults aged 20–79 years will be living with diabetes. Thus, while the world population is estimated to grow 20 % over this period, the number of subjects with diabetes is estimated to increase by 46 %^(1)^. Diabetes aetiology is multifactorial with a number of non-modifiable factors, such as family history (genotype) and age, and several modifiable factors, including those related to lifestyle, that contribute to the onset of the disease^(2)^. Therefore, prevention of T2D through the promotion of a healthy lifestyle in the population is a very important public health strategy. Amongst lifestyle factors, diet plays an important role^(3–5)^, and because analyses of single nutrients or foods cannot account for complex interactions, the assessment of dietary patterns has emerged as the most appropriate approach to examine the association between diet and T2D^(6)^.

Plant-based dietary patterns have gained significant attention in recent years for their potential to prevent or manage several chronic diseases such as cancer, neurodegenerative, cardiovascular and metabolic diseases^(7–13)^. Plant-based diets constitute a variety of dietary patterns that emphasise intake of foods derived from plant sources coupled with a lower consumption or the exclusion of animal products. Vegetarian diets form a subset of plant-based diets, which may exclude the consumption of some or all forms of animal foods^(14)^. Indeed, vegetarian diets are classified according to the patterns of elimination of food groups such as fish, eggs and/or dairy from diet (i.e. pesco-, ovo-, lacto- and lacto-ovo-vegetarians, respectively). The vegan diet is the most restrictive as it excludes all animal-based products, including fish, dairy, cheese and eggs.

The potential beneficial effect of dietary restrictions for animal-derived foods on the incidence of T2D is yet to be confirmed and characterised. Depending on the dietary definition, vegetarian patterns may exclude not only some animal products such as red and processed meats, which are positively associated with T2D risk^(15,16)^, but also others foods, such as dairy products and fish, which are potentially beneficial for human health, depending on the amount and their composition^(17–20)^. A meta-analysis of observational studies^(21)^ suggests an inverse association between every vegetarian dietary pattern and risk of T2D, although the study was limited by the inclusion of twelve cross-sectional studies and only two prospective studies, both of which were conducted in a selected population (U.S. Seventh-day Adventists). Therefore, it is still difficult to draw conclusions about the effectiveness of plant-based dietary patterns in the primary prevention of T2D. Moreover, several earlier studies on vegetarian diets and T2D have categorised study populations dichotomously into participants who do or do not consume some or all animal foods, thus preventing a comprehensive assessment of this potential association.

An important issue from both a nutritional and public health perspective, however, is whether a concomitant decrease in animal food intake and a higher adherence to a plant-rich diet lowers the risk of T2D. In this context, ‘plant-based diet index’ (PDI) is a new parameter that has recently been adopted to evaluate dietary habits with a focus on plant food intake^(22)^. An important consideration that needs to be taken into account is the fact that not all plant foods are necessarily healthy in relation to T2D, as well as other health endpoints. Several plant foods, such as fruits, vegetables, whole grains and legumes, are favourable for the prevention of T2D^(23–25)^, but other plant foods, such as refined grains, sweets and sugar-sweetened beverages, have undesirable effects on the development of T2D^(26–30)^. Thus, three plant-based diet indices were developed to differentiate the quality of plant-based foods in a person’s diet: overall PDI, healthy PDI (hPDI) and unhealthy PDI (uPDI) in order to consider whether they have a different impact on health outcomes. A recent dose–response meta-analysis^(13)^ used data from five prospective studies (Nurses’ Health Study, Nurses’ Health Study 2, Health Professionals Follow-Up Study, Singapore Chinese Health Study and Rotterdam Study) and showed a significant inverse and linear association between overall PDI and risk of T2D. However, this study did not perform a quantitative dose–response assessment stratified by adherence to hPDI or uPDI between increasing adherence and risk of diabetes. Conversely, a subsequent review reported an inverse association between PDI and hPDI with T2D risk^(31)^. However, it included a lower number of studies, and the dose–response analysis was implemented using a different model, and it was also not performed for uPDI.

The aim of this systematic review is to assess how adherence to vegan, vegetarian and plant-based diets, with separate consideration of healthy and unhealthy plant-based diets, is associated with risk of T2D, and to comprehensively characterise the pattern of the association taking advantage of the availability of newly published studies and advances in statistical techniques to model the dose–response relationship between investigated dietary indices and T2D.

## Methods

### Protocol registration

We followed the Preferred Reporting Items for Systematic Reviews and Meta-Analysis 2020 guidance^(32)^ to perform this review, and we registered the systematic review in PROSPERO database (no. CRD42022382022).

### Literature search and screening

We performed online literature searches in PubMed/MEDLINE and EMBASE databases up until 10 July 2025, by using the MeSH terms and keywords related to ‘plant-based diet’, ‘vegetarian diet’, ‘vegan diet’ and ‘type 2 diabetes’. Details about the search terms are reported in online Supplementary Table S1. We also used citation chasing techniques to identify relevant studies through screening of reference lists as well as backwards and forward citations of included studies^(33)^. We imported retrieved articles into Rayyan online application and duplicates were removed. Two authors (AM and TF) independently screened publication titles and abstracts and evaluated full-text publications for inclusion in the review. In case of disagreement, both authors performed a second review of the full text to determine eligibility for inclusion through a consensus-based discussion. If the two authors still disagreed, a third author (MV) was sought to resolve disagreement.

### Eligibility criteria and study selection

We defined the following study inclusion criteria according to the Population, Exposure, Comparison, Outcome and Study Design (PECOS) statement: (1) adult population (not pregnant women); (2) adherence to a plant-based, vegetarian or vegan diet; (3) subjects with low adherence to investigated dietary patterns; (4) assessment of T2D risk in relationship to plant-based, vegetarian or vegan diet adherence (outcome assessment may be based on clinical data related to the diagnosis or treatment of T2D, or on biochemical data after blood sampling) and (5) observational study design (cohort, cross-sectional or case–control). We did not apply any language restrictions. When necessary, we also contacted authors of included studies to retrieve additional information for data analysis when not published in the report.

### Risk of bias assessment

We assessed the quality of included studies using the Risk of Bias for non-randomised studies of exposures tool^(34)^. Seven domains were considered including: (1) bias due to confounding; (2) bias in selecting participants in the study; (3) bias in exposure classification; (4) bias due to departures from intended exposures; (5) bias due to missing data; (6) bias in outcome measurement and (7) bias in the selection of reported results. Online Supplementary Table S2 reports criteria for risk of bias evaluation. Two authors (AM and TF) performed the assessment, with any disagreements resolved by consultation with a third author (MV).

### Data extraction

We (AM and SP) extracted the following data from eligible studies using a standardised spreadsheet in Excel software: (1) study type; (2) first author name; (3) publication year; (3) country; (4) period of observation; (5) follow-up period; (6) type of exposure assessment; (5) outcome of interest; (6) estimation unit of adherence; (7) exposure categories; (8) dose for each category of exposure (for studies that used dietary indices); (8) number of cases with T2D; (9) sample size at baseline, overall and divided by exposure category; age (10) and sex (11) of participants at baseline; (12) risk estimates, either hazard ratio (HR), odds ratio (OR), and risk ratio (RR), with their 95 % confidence interval (CI) and covariates from the most adjusted multivariable model. For studies that used dietary indices, we used the risk estimate that compared the highest with the lowest percentiles, which represent the best (highest percentile) and poorest (lowest percentile) adherence to the plant-based dietary pattern. For studies that compared an *a priori* defined dietary pattern, we considered the study risk estimates comparing diets that are most restrictive of animal-based foods (e.g. vegan and vegetarian diets) with the least restrictive, such as omnivorous diet.

### Data analysis

First, we performed a forest-plot meta-analysis comparing vegetarian pattern/plant-based diet *v*. non-vegetarian dietary pattern and risk of T2D, using the non-vegetarian pattern as reference and comparing the highest *v*. the lowest category of exposure.

We then assessed the shape of the association between plant-based diet exposure and risk of T2D using a dose–response meta-analysis based on the one-stage approach^(35,36)^. In this approach, we used the mean/median levels or the midpoint of each exposure category, depending on data availability, and if the highest and the lowest exposure boundaries were ‘open’, a 20 % higher or lower value from the closest cutpoint^(37–39)^. We carried out this analysis by using a restricted cubic spline with three knots at fixed cutpoints (tenth, fiftieth and ninetieth percentiles) and a restricted maximum likelihood random effects model^(40,41)^. We used Stata-SE software (v19.0, Stata Corp., 2025) for all data analyses, specifically the ‘meta’, ‘mkspline’ and ‘drmeta’ routines.

### Subgroup and sensitivity analyses

We stratified all analyses using definitions of plant-based diets. Specifically, for vegetarian diets, analyses were stratified by type of vegetarianism (e.g. vegan and lacto-ovo-vegetarian), while for studies that defined adherence to plant-based dietary patterns using plant-based dietary indices, the results were divided by index type, that is, overall PDI, hPDI and uPDI. Specifically, hPDI emphasises consumption of vegetables, legumes and whole grains, while uPDI emphasises the intake of refined grains, sweets and sugar-sweetened beverages of plant origin^(14)^. Whenever possible, we also stratified the data by geographic region (namely Asian and Western countries, to account for differences in ethnic origin). We eventually performed sensitivity analyses by restricting the assessment to studies with cohort design only, with a duration of follow up equal to or above 10 years and excluding studies at high risk of bias.

### Heterogeneity and small study bias assessment

We assessed heterogeneity among included studies using the τ^2^, I^2^ and H^2^ statistics in the forest plots^(42)^ when comparing vegetarian/non-vegetarian patterns. For the dose–response analysis, we assessed the influence of variation across included studies with the graphical overlay of study-specific trends using predicted curves in the dose–response analysis risk^(43,44)^. Finally, we assessed publication bias with the presence of small-study effects through graphical presentation in funnel plots and Egger’s test^(45)^, and the trim-and-fill analysis^(46)^.

## Results

### Study selection

**Figure 1.**
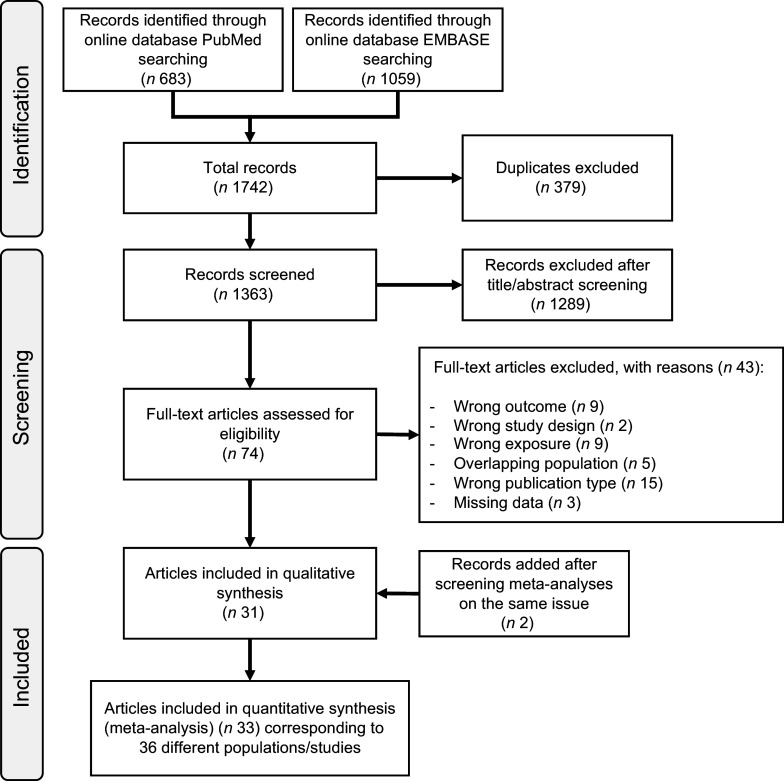
Flow chart of systematic literature search through 10 July 2025.

The Preferred Reporting Items for Systematic Reviews and Meta-Analysis flow chart of the literature search is presented in Figure 1. We retrieved 1363 articles after removal of duplicates, and we excluded 1289 articles after title and abstract screening. After full-text assessment of the remaining seventy-four articles, we further excluded forty-three articles because the outcome was not T2D (*n* 9); the exposure assessment did not include plant-based diet (*n* 9); the study design was based on a Mendelian randomisation method (*n* 2); some data were missing (*n* 3); the publication type (*n* 15) was wrong or there was a population overlap with some of the studies included (*n* 5). Two articles were added through backwards citation searching of included studies.

### Characteristics of included studies

Summary characteristics of the thirty-three articles eventually included in this review are reported in Table 1 ^(47–79)^. One article^(70)^ included three studies (Nurses’ Health Study, Nurses’ Health Study 2 and Health Professionals Follow-Up Study) and another^(61)^ included two cohorts (Centre for Cardiometabolic Risk Reduction in South-Asia and National Health and Nutrition Examination Survey), resulting in a total of thirty-six included studies. Overall, studies were published between 1999 and 2025, with most from Asia (*n* 14), followed by North America (*n* 13), Europe (*n* 7) and Central America (*n* 2). Of the thirty-six studies included in our analysis, nineteen are cohort^(49,50,52,53,55–57,62–65,67,69,70,72,73,75)^ and seventeen cross-sectional^(47,48,51,54,58–61,66,68,71,74,76–79)^. Regarding prospective studies, the duration of follow-up ranged from 2 to more than 20 years.

**Table 1.** Characteristics of the included studies divided by design (cohort and cross-sectional)

Reference	Country	Population characteristics/Study name	Study design and period	Exposure assessment	Exposure (index, category, mean/median value)			DM risk estimate	95 % CI	Outcome assessment	Adjustment factors
Cohort											
Bhupathiraju *et al.* ^(49)^	USA	Mediators of Atherosclerosis in South Asians Living in America (MASALA) Study	prospective cohort 2020–2018	Validated FFQ	PDI	Q1	51·10	Ref (OR)		Use of a glucose-lowering medication, FPG ≥ 126 mg/dl, and/or 2 h-PCG ≥ 200 mg/dl	Age, sex, study site, education, smoking status, alcohol, family history of diabetes, years lived in the USA, exercise, total energy, diabetes medication use, cholesterol-lowering medication use, hypertension medication use, sum of cultural traditional measures and BMI
						Q2	58·60	0·88	0·35, 2·24		
						Q3	63·80	0·84	0·33, 2·15		
						Q4	71·10	0·63	0·21, 1·91		
					hPDI	Q1	50·10	Ref (OR)			
						Q2	59·20	0·64	0·27, 1·50		
						Q3	65·40	0·62	0·25, 1·57		
						Q4	73·20	0·53	0·21, 1·35		
					uPDI	Q1	51·90	Ref (OR)			
						Q2	59·20	0·93	0·36, 2·36		
						Q3	63·90	1·54	0·62, 3·83		
						Q4	70·40	1·32	0·51, 3·47		
Boonpor *et al.* ^(50)^	UK	UK Biobank prospective cohort study	Prospective cohort 2006–2017	Validated FFQ	Meat eater			Ref (HR)		At baseline: self-reported diagnosis or in previous primary care, HbA1c ≥ 6·5 %. Incident type 2 diabetes was ascertained from prospective linkage to primary care records data.	Age, sex, deprivation, alcohol, smoking, sedentary time, sleep time and type of physical activity and BMI
					Vegetarian (lacto-ovo vegetarian)			1·15	0·90, 1·47		
Chen *et al.* ^(52)^	Singapore	Singapore Chinese Health Study (SCHS)	Prospective cohort 1993–2010	Validated FFQ	PDI	Q1	32·0	Ref (HR)		Self-report of a physician’s diagnosis of diabetes. The validity of diabetes reporting was assessed through linkage with a nationwide hospital-based discharge database or administration of a supplementary questionnaire about symptoms, diagnostic tests and hypoglycaemic therapy in a subsample of participants who reported physician-diagnosed diabetes, and through measure of HbA1c in blood samples among participants who did not report a diagnosis of diabetes	Age, sex, dialect group, year of baseline interview (1993–1995 or 1996–1998), energy intake, BMI, physical activity, education, smoking and self-reported history of physician-diagnosed hypertension
						Q2	36·0	0·96	0·87, 1·05		
						Q3	39·0	0·92	0·85, 1·01		
						Q4	42·0	0·87	0·79, 0·95		
						Q5	47·0	0·83	0·76, 0·92		
					hPDI	Q1	38·0	Ref (HR)			
						Q2	42·0	0·93	0·85, 1·01		
						Q3	45·0	0·93	0·85, 1·01		
						Q4	48·0	0·82	0·75, 0·90		
						Q5	52·0	0·81	0·75, 0·89		
Chen *et al.* ^(53)^	Netherlands	Rotterdam Study (RS) (three sub-cohorts)	Prospective cohort RS-I:1989–2012 RS-II:2000–2012 RS-III:2006–2012	Validated FFQ	PDI	Q1	40	HR per 10 units higher score on the plant-based dietary index: 0·87 (0·79, 0·99)		Information on T2D was collected from general practitioners’ records, pharmacies’ databases and follow-up examinations. T2D was identified according to WHO criteria: a FPG ≥ 126 mg/dl, a non-fasting blood glucose concentration of ≥ 200 mg/dl (when fasting samples were unavailable), or the use of blood glucose-lowering drugs or dietary treatment and registration of the diagnosis diabetes. All possible cases of T2D were formally judged by two independently working study physicians or, in case of disagreement, by an endocrinologist.	Energy intake, sex, age, Rotterdam Study sub-cohort (RS-I, -II, or -III), education, smoking status, family history of diabetes, physical activity, food supplement use and BMI
						Q2	46				
						Q3	50				
						Q4	53				
						Q5	59				
Chiu *et al.* ^(55)^	Taiwan	The Tzu Chi Health Study (TCHS)	Prospective cohort 2007–2016	Self-reported	Non-vegetarian			Ref (HR)		At baseline, participants with diabetes as reported in the baseline questionnaire or with FPG ≥ 126 mg/dl were excluded. Incident cases of diabetes were identified if participants reported diabetes diagnosis at follow-up questionnaires, or if their HbA1C ≥ 6·5 %. Participants with only one fasting blood glucose ≥ 126 mg/dl but otherwise normal HbA1C were identified as possible diabetes cases. For these individuals, a physician co-author further reviewed their medical records to check if they had additional blood tests or prescriptions for diabetes medication to determine their diabetes status. Participants without further tests or available medical records were considered unconfirmed diabetes events and were excluded, but included as cases in a sensitivity analysis.	Age, sex, education, leisure time physical activities, family history of diabetes, follow-up methods, lipid medication and BMI
					Vegetarian (vegan, lacto-ovo vegetarian, lacto-vegetarian, ovo-vegetarian)			0·65	0·46, 0·92		
Choi *et al.* ^(56)^	USA	Coronary Artery Risk Development in Young Adults (CARDIA) Study	Prospective cohort 2005–2016	Validated CARDIA diet history questionnaire	APDQS	Q1	48	Ref (HR)		FPG ≥ 126 mg/dl, 2 h-PCG ≥ 200 mg/dl, HbA1c ≥ 6·5 %, and/or self-reported use of antidiabetic medications (per medication bottle brought to clinic).	Age, sex, race and total energy intake, parental history of diabetes, physical activity level, smoking status, highest grade of education achieved during follow-up and BMI
						Q2	63·5	0·97	0·69, 1·38		
						Q3	71·0	0·59	0·38, 0·91		
						Q4	78·5	0·57	0·35, 0·93		
						Q5	99·6	0·33	0·18, 0·61		
Flores *et al.* ^(57)^	USA	Boston Puerto Rican Health Study (BPRHS)	Prospective cohort 2004–2009	Validated FFQ	PDI	T1	48	Ref (HR)		FPG ≥ 126 mg/dl, HbA1c ≥ 6·5 %, or use of hypoglycaemic agents.	Age, sex, education, marital status, income to poverty ratio, total energy, smoking status, alcohol frequency, physical activity score, psychological acculturation score, depressive symptomatology score, BMI, hypertension, plasma cholesterol and antilipemic agents
						T2	54	0·86	0·54, 1·38		
						T3	61	0·79	0·48, 1·30		
					hPDI	T1	46	Ref (HR)			
						T2	53	0·79	0·48, 1·30		
						T3	61	0·54	0·31, 0·94		
					uPDI	T1	48	Ref (HR)			
						T2	55	1·68	1·00, 2·80		
						T3	62	1·19	0·66, 2·14		
Kim and Giovannucci^(62)^	Korea	Korean Genome and Epidemiology Study (KoGES)	Prospective cohort 2001–2016	Validated FFQ	PDI	Q1	42·87	Ref (HR)		FPG ≥ 126 mg/dl, self-report of a doctor’s diagnosis of T2D, use of oral hypoglycaemic drug or current treatment with insulin.	Age, sex, residence area, education, physical activity, cigarette smoking, alcohol intake, baseline BMI, total energy intake, family history of diabetes and history of hypertension at baseline
						Q2	48·52	0·92	0·75, 1·14		
						Q3	52·46	0·96	0·79, 1·17		
						Q4	56·78	0·87	0·70, 1·07		
						Q5	63·28	1·05	0·86, 1·28		
					hPDI	Q1	43·49	Ref (HR)			
						Q2	48·06	0·88	0·72, 1·07		
						Q3	50·98	0·80	0·65, 0·99		
						Q4	53·89	0·78	0·63, 0·95		
						Q5	58·51	0·81	0·66, 1·00		
					uPDI	Q1	42·04	Ref (HR)			
						Q2	47·59	0·99	0·81, 1·22		
						Q3	51·03	1·07	0·87, 1·31		
						Q4	54·39	1·01	0·82, 1·25		
						Q5	60·08	1·16	0·92, 1·46		
Koloverou *et al.* ^(63)^	Greece	ATTICA Cohort Study	Prospective cohort 2001–2012	Validated FFQ	Non-vegetarian dietary pattern			Ref (OR)		Diagnosis was conducted according to American Diabetes Association criteria: FPG > 125 mg/dl or the use of antidiabetic agents and/or insulin.	Sex, family history of diabetes, waist circumference and smoking status
					Semi-vegetarian-like dietary pattern			< 45a: 1·89	0·85, 4·18		
								45–55a: 0·60	0·34, 1·07		
								> 55a: 0·19	0·02, 2·03		
Laouali *et al.* ^(64)^	France	Etude Epidémiologique auprès de femmes de la Mutuelle Générale de l’Education Nationale (E3N)	Prospective cohort 1993–2014	Validated FFQ	PDI	Q1	43·13	Ref (HR)		Before 2004: self-reported diagnosis of diabetes, use of antidiabetic drugs, and hospitalisations for diabetes. All potential cases were contacted and asked to answer a diabetes specific questionnaire. For T2D validation, at least 1 of the following: (1) FPG ≥ 126 mg/dl or random glucose ≥ 200 mg/dl at diagnosis, (2) use of a glucose lowering medication, and (3) most recent values of FPG ≥ 126 mg/dl or HbA1c concentration ≥ 7·0 % in the diabetes-specific questionnaire. After 2004: drug reimbursement insurance database	Age, family history of diabetes, educational level, hypercholesterolaemia, hypertension, smoking status, physical activity, energy intake and BMI
						Q2	48·61	0·88	0·80, 0·97		
						Q3	52·00	0·82	0·74, 0·91		
						Q4	55·39	0·72	0·64, 0·80		
						Q5	60·79	0·71	0·63, 0·79		
					hPDI	Q1	45·85	Ref (HR)			
						Q2	51·04	0·99	0·89, 1·10		
						Q3	54·50	0·85	0·77, 0·94		
						Q4	57·94	0·82	0·73, 0·92		
						Q5	63·16	0·74	0·67, 0·83		
					uPDI	Q1	44·63	Ref (HR)			
						Q2	51·16	1·05	0·95, 1·16		
						Q3	55·00	0·94	0·84, 1·05		
						Q4	58·40	0·91	0·81, 1·02		
						Q5	64·14	0·99	0·89, 1·11		
Lv *et al.* ^(65)^	China	China Health and Nutrition Survey (CHNS)	Prospective cohort 1997–2015	3rd 24 h food recalls	PDI	Q1	37	Ref (HR)		Survey 2009: FPG ≥ 126 mg/dl, HbA1c ≥ 6·5 %, or self-reported diagnosis of T2D or on hypoglycaemic medication Survey 2015: self-reported diabetes or taking hypoglycaemic medication	Age, sex, BMI, region, urbanisation index, educational level, physical activity, baseline hypertension, smoking status, alcohol intake and total energy intake
						Q2	43	0·71	0·58, 0·86		
						Q3	48	0·57	0·46, 0·70		
						Q4	53	0·46	0·36, 0·58		
						Q5	59	0·34	0·26, 0·46		
					hPDI	Q1	45	Ref (HR)			
						Q2	49	0·89	0·72, 1·09		
						Q3	52	0·73	0·59, 0·89		
						Q4	55	0·76	0·62, 0·94		
						Q5	59	0·81	0·65, 0·99		
					uPDI	Q1	43	Ref (HR)			
						Q2	48	1·32	1·07, 2·60		
						Q3	51	1·50	1·21, 1·85		
						Q4	54	1·70	1·38, 2·10		
						Q5	59	2·18	1·75, 2·73		
Papier *et al.* ^(67)^	UK	European Prospective Investigation into Cancer and Nutrition (EPIC)-Oxford study	Prospective cohort 1993–2016	Validated FFQ	Regular meat eater			Ref (HR)		At baseline were excluded participants self-reporting prior diabetes. Diabetes status was ascertained through health record linkage. The data available included information on hospital admissions and deaths. Any diagnosis of diabetes (i.e. not necessarily the primary diagnosis) or mention of diabetes among the causes of death or contributory conditions was considered for this study.	Age, education, Townsend deprivation index, ethnicity, smoking, alcohol intake, physical activity and BMI
					Vegetarian (not consuming meat or fish but consuming dairy products or eggs)			0·89	0·75, 1·05		
					Vegan			0·99	0·66, 1·48		
Satija *et al.* ^(70)^	USA	Nurses’ Health Study (NHS)	Prospective cohort 1984–2012	Validated FFQ	PDI	Q1	45·5	Ref (HR)		Self-report of a physician’s diagnosis of diabetes confirmed by a supplementary questionnaire with established validity. Only confirmed cases that met ≥ 1 of the following criteria were included: (a) ≥ 1 classic symptoms plus FPG ≥ 140 mg/dl or random blood glucose ≥ 200 mg/dl; (b) no symptoms, but raised blood glucose levels (i.e. FPG ≥ 140 mg/dl or random blood glucose ≥ 200 mg/dl or 2 h-PCG ≥ 200 mg/dl) on two different occasions; (c) treatment with hypoglycaemic drugs. The threshold for FPG was changed to ≥ 126 mg/dl starting in 1998; HbA1c ≥ 6·5 % was further added to the diagnosis criteria starting in 2010	Age, smoking status, physical activity, alcohol intake, multivitamin use, family history of diabetes, margarine intake, energy intake, baseline hypertension, baseline hypercholesterolaemia, menopause status and postmenopausal hormone use and BMI
						Q2	48·8	1·00	0·91, 1·10		
						Q3	50·8	0·93	0·85, 1·03		
						Q4	52·4	0·99	0·90, 1·09		
						Q5	54·0	0·92	0·83, 1·02		
						Q6	55·2	0·87	0·78, 0·96		
						Q7	56·7	0·88	0·80, 0·98		
						Q8	58·2	0·81	0·73, 0·90		
						Q9	60·2	0·85	0·76, 0·94		
						Q10	63·6	0·83	0·74, 0·93		
					hPDI	Q1	44·3	Ref (HR)			
						Q2	48·2	0·98	0·89, 1·06		
						Q3	50·6	0·87	0·79, 0·95		
						Q4	52·5	0·82	0·75, 0·90		
						Q5	54·0	0·77	0·70, 0·85		
						Q6	55·8	0·79	0·72, 0·87		
						Q7	57·5	0·80	0·72, 0·88		
						Q8	59·3	0·73	0·65, 0·80		
						Q9	61·6	0·70	0·63, 0·78		
						Q10	65·5	0·60	0·54, 0·68		
Satija *et al.* ^(70)^	USA	Nurses’ Health Study 2 (NHS2)	Prospective cohort 1991–2011	Validated FFQ	PDI	Q1	45·3	Ref (HR)		Self-report of a physician’s diagnosis of diabetes confirmed by a supplementary questionnaire with established validity. Only confirmed cases that met ≥ 1 of the following criteria were included: (a) ≥ 1 classic symptoms plus FPG ≥ 140 mg/dl or random blood glucose ≥ 200 mg/dl; (b) no symptoms, but raised blood glucose levels (i.e. FPG ≥ 140 mg/dl or random blood glucose ≥ 200 mg/dl or 2 h-PCG ≥ 200 mg/dl) on two different occasions; (c) treatment with hypoglycaemic drugs. The threshold for FPG was changed to ≥ 126 mg/dl starting in 1998; HbA1c ≥ 6·5 % was further added to the diagnosis criteria starting in 2010	Age, smoking status, physical activity, alcohol intake, multivitamin use, family history of diabetes, margarine intake, energy intake, baseline hypertension, baseline hypercholesterolaemia, menopause status and postmenopausal hormone use, oral contraceptive use and BMI
						Q2	48·8	0·98	0·88, 1·09		
						Q3	51·0	0·88	0·78, 0·98		
						Q4	52·5	0·82	0·73, 0·92		
						Q5	54·0	0·94	0·84, 1·06		
						Q6	55·3	0·88	0·78, 0·99		
						Q7	57·0	0·97	0·86, 1·09		
						Q8	58·7	0·86	0·75, 0·97		
						Q9	61·0	0·91	0·80, 1·03		
						Q10	64·3	0·83	0·72, 0·95		
					hPDI	Q1	44·0	Ref (HR)			
						Q2	48·0	1·05	0·94, 1·17		
						Q3	50·3	0·99	0·88, 1·10		
						Q4	52·3	1·00	0·89, 1·12		
						Q5	54·0	0·92	0·82, 1·03		
						Q6	55·8	0·93	0·82, 1·04		
						Q7	57·4	0·93	0·83, 1·05		
						Q8	59·2	0·85	0·75, 0·96		
						Q9	61·7	0·86	0·76, 0·98		
						Q10	66·0	0·77	0·67, 0·89		
Satija *et al.* ^(70)^	USA	Health Professionals Follow-Up Study (HPFS)	Prospective cohort 1986–2010	Validated FFQ	PDI	Q1	45·0	Ref (HR)		Self-report of a physician’s diagnosis of diabetes confirmed by a supplementary questionnaire with established validity. Only confirmed cases that met ≥ 1 of the following criteria were included: (a) ≥ 1 classic symptoms plus FPG ≥ 140 mg/dl or random blood glucose ≥ 200 mg/dl; (b) no symptoms, but raised blood glucose levels (i.e. FPG ≥ 140 mg/dl or random blood glucose ≥ 200 mg/dl or 2 h-PCG ≥ 200 mg/dl) on two different occasions; (c) treatment with hypoglycaemic drugs. The threshold for FPG was changed to ≥ 126 mg/dl starting in 1998; HbA1c ≥ 6·5 % was further added to the diagnosis criteria starting in 2010	Age, smoking status, physical activity, alcohol intake, multivitamin use, family history of diabetes, margarine intake, energy intake, baseline hypertension, baseline hypercholesterolaemia and BMI
						Q2	48·5	0·95	0·83, 1·09		
						Q3	50·6	0·92	0·80, 1·06		
						Q4	52·3	0·92	0·80, 1·06		
						Q5	54·0	0·87	0·75, 1·00		
						Q6	55·5	0·79	0·68, 0·92		
						Q7	57·0	0·84	0·72, 0·98		
						Q8	58·6	0·74	0·63, 0·87		
						Q9	61·0	0·85	0·72, 0·99		
						Q10	64·4	0·70	0·59, 0·83		
					hPDI	Q1	43·3	Ref (HR)			
						Q2	47·3	0·93	0·81, 1·07		
						Q3	50·0	0·87	0·76, 1·01		
						Q4	52·0	0·81	0·70, 0·94		
						Q5	53·8	0·82	0·70, 0·95		
						Q6	55·3	0·78	0·67, 0·91		
						Q7	57·0	0·75	0·64, 0·87		
						Q8	59·2	0·70	0·59, 0·82		
						Q9	61·8	0·66	0·56, 0·77		
						Q10	66·0	0·65	0·55, 0·77		
Sullivan *et al.* ^(69)^	USA	Atherosclerosis Risk in Communities (ARIC) Study	Prospective cohort 1987–2021	Validated FFQ	PDI	Q1	44	Ref (HR)		Self-reported diagnosis by a health care provider, or current diabetes medication use, or FPG ≥ 126 mg/dl, or non-fasting serum glucose ≥ 200 mg/dl	Age, sex, race-centre, total energy intake, education, income, smoking status, physical activity, margarine intake, alcohol intake and BMI
						Q2	49	1·02	0·93, 1·11		
						Q3	52	0·92	0·84, 1·00		
						Q4	55	0·97	0·88, 1·07		
						Q5	59	0·93	0·85, 1·03		
					hPDI	Q1	43	Ref (HR)			
						Q2	48	0·96	0·88, 1·05		
						Q3	51	0·89	0·81, 0·98		
						Q4	54	0·94	0·85, 1·03		
						Q5	60	0·92	0·83, 1·02		
					uPDI	Q1	43	Ref (HR)			
						Q2	48	0·93	0·84, 1·02		
						Q3	51	0·97	0·89, 1·07		
						Q4	55	1·08	0·98, 1·19		
						Q5	60	1·02	0·92, 1·13		
Thompson *et al.* ^(72)^	UK	UK Biobank prospective cohort study	Prospective cohort 2009–2021 (England) 2009–2021 (Scotland) 2009–2016 (Wales)	Web-based 24-h dietary assessment tool (the validated OxfordWebQ)	hPDI	Q1	47·70	Ref (HR)		Incident T2D: hospital inpatient data on admissions and diagnoses	Sex, BMI, waist circumference, ethnicity, physical activity, smoking status, alcohol intake, education, energy intake, polypharmacy index, multimorbidity index, Townsend deprivation index, family history of diabetes, prevalent hypercholesterolaemia, prevalent hypertension, menopausal status, Polygenetic risk scores for T2DM and number of completed dietary assessments
						Q2	53·70	0·84	0·76, 0·93		
						Q3	57·60	0·82	0·73, 0·91		
						Q4	63·40	0·76	0·68, 0·85		
					uPDI	Q1	46·90	Ref (HR)			
						Q2	52·40	1·15	1·03, 1·29		
						Q3	56·20	1·24	1·11, 1·39		
						Q4	61·50	1·37	1·22, 1·53		
Tonstad *et al.* ^(73)^	USA, Canada	Adventist Health Study 2 (AHS-2)	Prospective cohort 2002–2007	Validated FFQ	Non-vegetarian			Ref (OR)		Self-reported diagnosis. A list of participants reporting diabetes was randomly selected for verification of the self-reported development of diabetes	Age, sex, ethnicity, education, income, TV watching, physical activity, hours of sleep, alcohol consumption, smoking and BMI
					Vegan			0·38	0·24, 0·62		
					Lacto-ovovegetarian			0·62	0·50, 0·76		
					Pesco-vegetarian			0·79	0·58, 1·09		
					Semi-vegetarian			0·49	0·31, 0·76		
Vang *et al.* ^(75)^	USA	Adventist Mortality Study (AMS) and Adventist Health Study (AHS)	Prospective cohort 1960–1976	Validated FFQ	Long-term vegetarian (not consuming meat)			Ref (OR)		Self-reported diagnosis	Age, sex, BMI and weight change
					Long-term non-vegetarian			1·38	1·06, 1·80		
Cross-sectional											
Agrawal *et al.* ^(47)^	India	India’s third National Family Health Survey (NFHS-3)	Cross-sectional 2005–2006	Food propensity questionnaire (FPQ)	Non-vegetarian			Ref (OR)		Self-reported diagnosis	Age, sex, education, household wealth, rural/urban residence, religion, caste, smoking, alcohol use, television watching and BMI
					Vegan			0·91	0·61, 1·36		
					Lacto-ovovegetarian			0·70	0·51, 0·96		
					Pesco-vegetarian			1·15	0·85, 1·54		
Bharati *et al.* ^(48)^	South India	Rural and urban field practice area of Mahatma Gandhi Medical College and Research Institute, Puducherry	Cross-sectional 2007–2008	Pre-designed and pre-tested questionnaire (type of food, dietary habit)	Vegetarian (not specified)			Ref (OR)		FPG ≥ 126 mg/dl and not on treatment (2006 WHO recommendation used for diagnostic criteria for the diagnosis of diabetes mellitus, WHO/International Diabetes Federation)	Age, residence, education, tobacco addiction, BMI, waist-hip ratio and total blood cholesterol
					Non-vegetarian			5·74	1·35, 24·40		
Brathwaite *et al.* ^(51)^	Barbados	Barbadian Seventh-Day-Adventists	Cross-sectional 1999	Self-reported	Self-reported vegetarian (not specified)			Ref (OR)		FPG ≥ 140 mg/dl or self-reported physician’s diagnosis of diabetes (according to the WHO criteria)	Crude
					Non Self-reported vegetarian			1·27	077, 2·10		
Chiu *et al.* ^(54)^	Taiwan	The Tzu Chi Health Study	Cross-sectional 2007–2009	Validated FFQ	Omnivorous diet			Ref (OR)		Self-reported history of diabetes ascertained from the baseline medical history questionnaire or a FPG ≥ 126 mg/dl. Two physicians subsequently confirmed the self-reported diabetes with the electronic medical records in Tzu Chi hospitals. For those who did not have medical records, the physicians made telephone calls to confirm with the participants about their diabetes diagnosis. Participants who did not self-report a history of diabetes but had a FPG ≥ 126 mg/dl were regarded as having diabetes if one of the following criteria was further confirmed in medical record or with the participants in a telephone follow-up: (1) physician diagnosis of diabetes; (2) prescription of diabetes medication; (3) an additional fasting plasma glucose ≥ 126 mg/dl and (4) an additional HbA1C ≥ 6·5 %.	Age, BMI, family history of diabetes, education, leisure time physical activity (LTPA), smoking (men only) and alcohol (males only)
					Vegetarian diet (completely avoiding meat, fish, and all animal flesh)			M: 0·49	0·28, 0·89		
								Pre-menopausal W: 0·26	0·06, 1·21		
								Menopausal W: 0·25	0·15, 0·42		
Fraser^(58)^	USA	Adventist Health Study (AHS)	Cross-sectional 1976–1988	Validated FFQ	Vegetarian (not eating fish, poultry, or meat)			Ref (OR)		Self-reported but confirmed by physicians’ diagnoses	Age
					Non-vegetarian			M: 1·97	1·56, 2·47		
								W: 1·93	1·65, 2·25		
Golebiowska *et al.* ^(59)^	Poland	National Test for Poles’ Health (NTPH)	Cross-sectional 2021–2022	Self-reported	Non-vegetarian diet			Ref (OR)		Self-reported diagnosis	Age, sex, education level, married, population of the place of residence, period of the study (during the pandemic), dairy-free meals and BMI
					Vegetarian diet (not specified)			0·73	0·55, 0·96		
Heidarzadeh-Esfahani *et al.* ^(60)^	Iran	Ravansar Non-Communicable Disease (RaNCD) cohort study	Cross-sectional 2014–2015	Validated FFQ	PDI	T1	47·13	Ref (OR)		FPG ≥ 126 mg/dl and/or treatment with antidiabetic medications	Age, sex, energy intake, physical activity, socioeconomic status
						T2	54·44	1·00	0·79, 1·26		
						T3	61·57	0·70	0·51, 0·96		
Jaacks *et al.* ^(61)^	South Asia	Centre for Cardiometabolic Risk Reduction in South-Asia (CARRS)	Cross-sectional 2011	Food propensity questionnaire (FPQ)	Non-vegetarian			Ref (OR)		FPG ≥ 126 mg/dl, HbA1c ≥ 6·5 %, or treatment of previously diagnosed diabetes with oral agents or insulin	Age, sex, education, tobacco, alcohol and city
					Vegetarian (vegan, lacto-vegetarian, lacto-ovovegetarian, pesco-vegetarian, semi-vegetarian)			1·04	0·86, 1·27		
Jaacks *et al.* ^(61)^	USA	National Health and Nutrition Examination Survey (NHANES)	Cross-sectional 2003–2006	Food propensity questionnaire (FPQ)	Non-vegetarian			Ref (OR)		FPG ≥ 126 mg/dl, HbA1c ≥ 6·5 %, or treatment of previously diagnosed diabetes with oral agents or insulin	Age, sex, education, tobacco and alcohol
					Vegetarian (vegan, lacto-vegetarian, lacto-ovovegetarian, pesco-vegetarian, semi-vegetarian)			0·75	0·29, 1·96		
Misra *et al.* ^(66)^	USA	Adult Asian Indians in the USA	Cross-sectional 2000–2002	Self-reported	Non-vegetarian			Ref (OR)		FBG ≥ 126 mg/dl (using capillary blood glucose measurement rather than serum glucose) or self-report of previously diagnosed DM.	Age, sex, income, access to care, tobacco use, acculturation, physical activity, diet change after immigration to the USA, healthy dietary habits, and family history of diabetes, HDL, blood pressure, CRP, homocysteine and lipoprotein(a)
					Vegetarian (lacto-vegetarian)			0·55	0·31, 0·99		
Ponzio *et al.* ^(68)^	Italy	Health and Use of Health Care in Italy	Cross-sectional 2004–2005	Self-reported	Non-vegetarian			Ref (OR)		Self-reported diagnosis	Age, sex, education, marital status, smoking, stroke, hypertension, obesity, self-reported health status and weight control.
					Vegetarian (not specified)			1·37	1·06, 1·75		
Shridhar *et al.* ^(71)^	India	Indian Migration Study (IMS)	Cross-sectional 2005–2007	Validated FFQ	Non-vegetarian			Ref (RR)		Diagnosis of diabetes made with the WHO FPG criterion of ≥ 126 mg/dl or report of a doctor’s diagnosis of diabetes.	Crude
					Vegetarian (not eating eggs, meat, fish and poultry)			0·94	0·81, 1·10		
Tonstad *et al.* ^(74)^	USA, Canada	Adventist Health Study 2 (AHS-2)	Cross-sectional 2002–2006	Validated FFQ	Non-vegetarian			Ref (OR)		Cases of diabetes were ascertained by asking whether a physician had ever diagnosed type 1 or type 2 diabetes and whether the respondent was treated for this in the last 12 months. A representative subgroup of study subjects participated in a calibration study and provided blood samples for measurement of FPG levels.	Age, sex, ethnicity, education, income, physical activity, television watching, sleep habits, alcohol use and BMI
					Vegan			0·51	0·40, 0·66		
					Lacto-ovovegetarian			0·54	0·49, 0·60		
					Pesco-vegetarian			0·70	0·61, 0·80		
					Semi-vegetarian			0·76	0·65, 0·90		
Yang *et al.* ^(76)^	China	Henan Rural Cohort Study	Cross-sectional 2015–2017	Validated FFQ	PDI	Q1	NR	Ref (OR)		According to the diagnostic criteria of the ADA, participants were defined as T2D if their FPG was ≥ 126 mg/dl or participants reported having previously been diagnosed with T2D and/or the use of insulin or blood glucose-lowering drugs.	Age, sex, education level, marital status, per capita monthly income, tobacco smoking, alcohol drinking, total energy intake, physical activity, hypertension, family history of diabetes and BMI
						Q2	NR	0·97	0·87, 1·08		
						Q3	NR	0·99	0·89, 1·09		
						Q4	NR	0·88	0·79, 0·98		
Yogal *et al.* ^(77)^	Nepal	Women in rural Nepal	Cross-sectional 2012–2013	Self-reported	Vegetarian (not specified)			Ref (OR)		Diabetes was diagnosed according to one of the following criteria: a positive history of diabetes, the use of antidiabetic medication or HbA1c of 6·5 %, American Diabetes Association·	Age
					Non-vegetarian			0·5	0·22, 1·16		
Zhang *et al.* ^(78)^	China	Suburban residents (Beijing)	Cross-sectional 2007	Standardised questionnaire (dietary habits)	Non-vegetarian			Ref (OR)		FPG ≥ 126 mg/dl or self-reported current treatment with antidiabetic medication (insulin or oral hypoglycaemic agents)	Age, sex, smoking, drinking and other potential confounders
					Vegetarian (not eating meat)			0·68	0·55, 0·86		
Zhang *et al.* ^(79)^	China	China Patient-Centered Evaluative Assessment of Cardiac Events Million Persons Project (MPP)-Shanxi Study	Cross-sectional 2017–2019	Validated FFQ	PDI	Q1	35·5	Ref (OR)		Diagnosis based on an FPG ≥ 126 mg/dl or the use of a hypoglycaemic drug.	Age, sex, tobacco smoking, alcohol drinking, physical activity, marital status, waist circumference, geographic region, occupation, education, household income, hypertension, dyslipidaemia and BMI
						Q2	45·5	0·90	0·83, 0·96		
						Q3	48	0·83	0·78, 0·89		
						Q4	55	0·82	0·76, 0·89		

BMI, body mass index; FPG, fasting plasma glucose; 2 h-PCG, 2-h post-challenge glucose concentration; HbA1c: glycosylated Hb; M, males; W, women; NR, not reported; T2D, type 2 diabetes.

Sixteen studies defined adherence to plant-based dietary patterns using a plant-based dietary index, fifteen of them calculated an overall PDI, eleven studies a hPDI and seven studies an uPDI. The remaining twenty studies defined adherence to vegetarian or vegan diets using *a priori*-defined dietary patterns. Eight studies evaluated specific vegetarian patterns, including vegan, lacto-ovo vegetarian, pesco-vegetarian and semi-vegetarian, while twelve did not stratify the analyses by type of vegetarianism. Specifically, in three studies, the term ‘vegetarian’ referred to a single group of participants adhering to any plant-based dietary patterns, namely vegan, lacto-ovo vegetarian, lacto-vegetarian and ovo-vegetarian^(55)^; and vegan, lacto-ovo vegetarian, lacto-vegetarian, pesco-vegetarian and semi-vegetarian in Centre for Cardiometabolic Risk Reduction in South-Asia and National Health and Nutrition Examination Survey cohorts^(61)^. In nine studies, no definition for vegetarian diet was reported.

Among the studies that assessed adherence using a plant-based dietary index, all but two provided data suitable for dose–response meta-analysis. In one study, exposure doses based on dietary habits assessment were not available^(76)^; another study did not provide the estimated risk of T2D for each exposure category^(53)^.

### Risk of bias assessment

Results of study quality assessment by risk of bias are reported in online Supplementary Table S3. Overall, fourteen of the included studies were judged at high risk of bias^(47,51,55,58,59,61,63,66,68,71,73–75,77)^, while five studies were at overall moderate risk of bias^(48,52,54,70,78)^. Concerning individual domains, six studies were considered at moderate risk of bias due to lack of control for BMI^(58,60,61,66,77,78)^ and additional two were considered at high risk of bias due to confounding, since they did not implement any multivariable model but reported crude data^(51,71)^. Seven studies were considered at moderate risk of selection bias, because participant selection was linked to a predominantly plant-based diet^(51,54,55,58,73–75)^. Regarding information bias, the risk of exposure misclassification was moderate for five studies^(47,48,51,61,78)^ because details about questionnaire validation were not specified, while it was high for five studies^(55,59,66,68,77)^ in which vegetarian status was self-reported. As regards bias in departure from intended exposure, six studies were at high risk of bias because exposure levels were not reported^(51,55,59,66,68,77)^. Two studies excluded participants due to missing data ≥ 20 %^(63,74)^. Outcome identification was based on self-report in six studies^(52,58,64,70,73,74)^, and additional five studies outcome assessment was based on self-report only without external validation, or was not based on international guidelines^(47,59,66,68,75)^, thus they were considered at high risk of bias. Overall, 58 % of studies were considered at moderate/high risk of bias. Considering high risk of bias only and division by study design, the proportion was much lower in cohort than cross-sectional studies, with respectively 21 % (4/19) and 62 % (10/16).

### Quantitative synthesis

**Figure 2.**
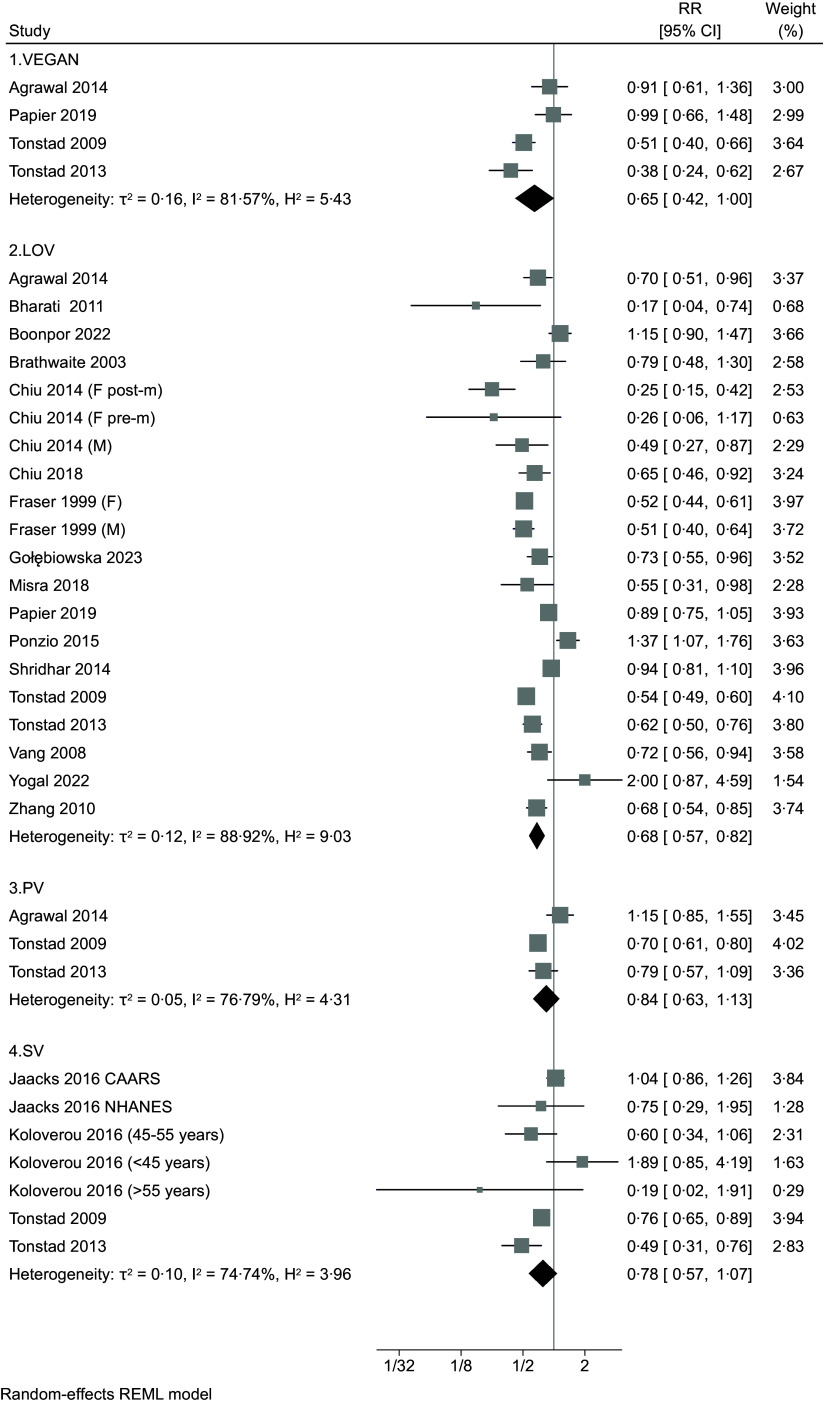
Forest plot of the included studies regarding risk of type 2 diabetes comparing the highest *v*. the lowest adherence to different plant-based dietary patterns (VEGAN: vegan diet; LOV: lacto-ovo-vegetarian diet; PV: pesco-vegetarian diet; SV: semi-vegetarian diet). RR: risk ratio. CI: confidence interval. The area of each grey square is proportional to the inverse of the variant of the estimated RR, and horizontal lines represent the 95 % CI. Black diamonds represent point estimates of overall RR for each group. The solid vertical line represents null effect (RR = 1).

**Figure 3.**
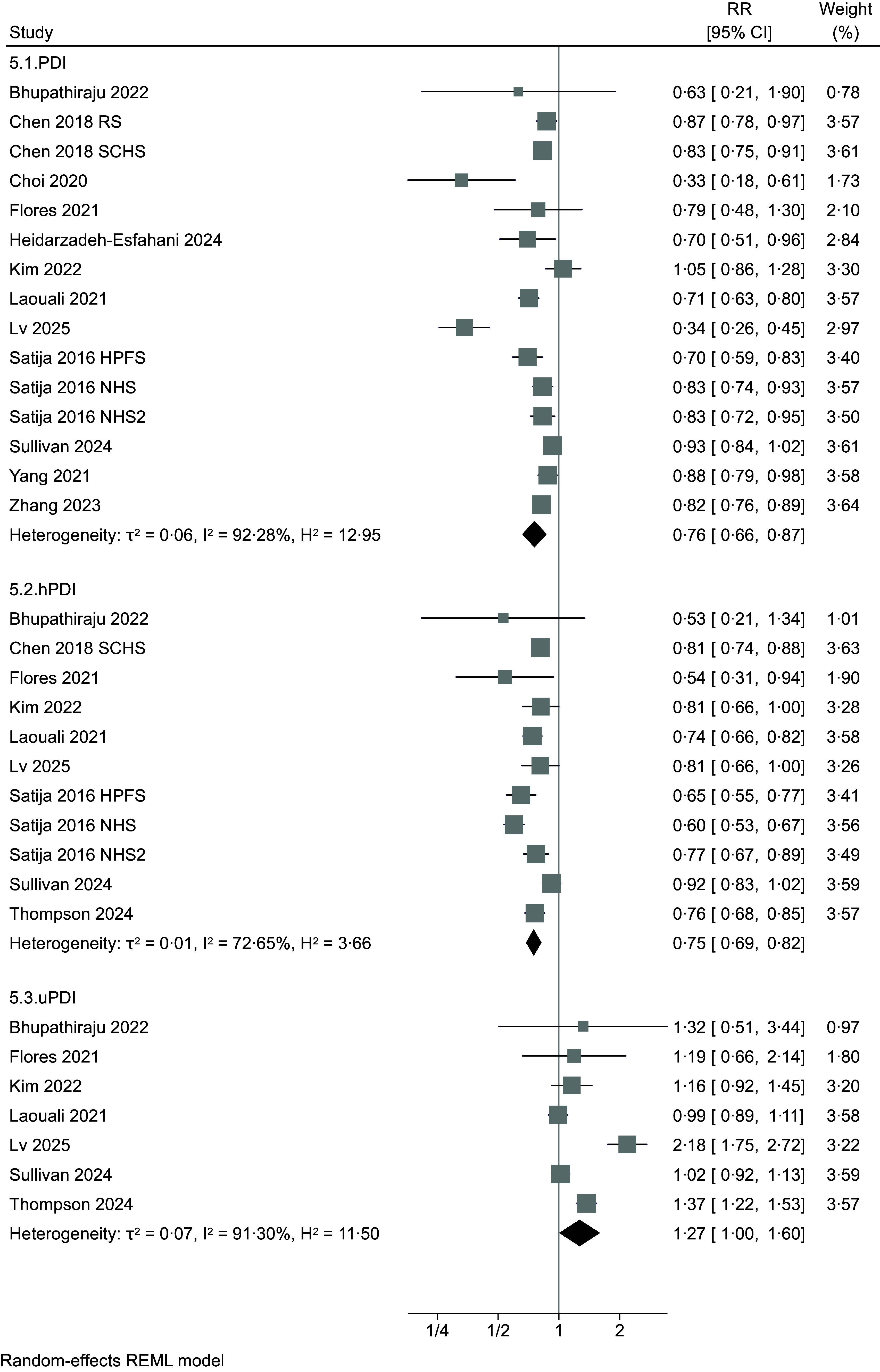
Forest plot of the included studies regarding risk of type 2 diabetes comparing the highest *v*. the lowest adherence to different plant-based dietary patterns (PDI: plant-based diet index; hPDI: healthy PDI; uPDI: unhealthy PDI) RR: risk ratio. CI: confidence interval. The area of each grey square is proportional to the inverse of the variant of the estimated RR, and horizontal lines represent the 95 % CI. Black diamonds represent point estimates of overall RR for each group. The solid vertical line represents null effect (RR = 1).

Forest plot analyses comparing the highest *v*. the lowest adherence to vegetarian/plant-based dietary patterns (Figure 2) showed a negative association with T2D independently from the type of pattern, with RR of 0·65 (95 % CI 0·42, 1·00, four studies, τ^2^ = 0·16, I^2^ = 81·57 %), 0·68 (95 % CI 0·57, 0·82, seventeen studies, τ^2^ = 0·12, I^2^ = 88·91 %), 0·84 (95 % CI 0·63, 1·13, three studies, τ^2^ = 0·05, I^2^ = 76·79 %) and 0·78 (95 % CI 0·57, 1·07, five studies, τ^2^ = 0·10, I^2^ = 74·74 %) for vegan, lacto-ovo-vegetarian, pesco-vegetarian and semi-vegetarian, respectively. For studies that defined adherence using a PDI (Figure 3), RR associated comparing extreme categories of intake was 0·76 (95 % CI 0·66, 0·87, fifteen studies, τ^2^ = 0·06, I^2^ = 92·28 %). When hPDI and uPDI are considered, RR were 0·75 (95 % 0·69, 0·82, eleven studies, τ^2^ = 0·01, I^2^ = 72·65 %) and 1·27 (95 % CI 1·00, 1·60, seven studies, τ^2^ = 0·07, I^2^ = 91·30 %), respectively.

**Figure 4.**
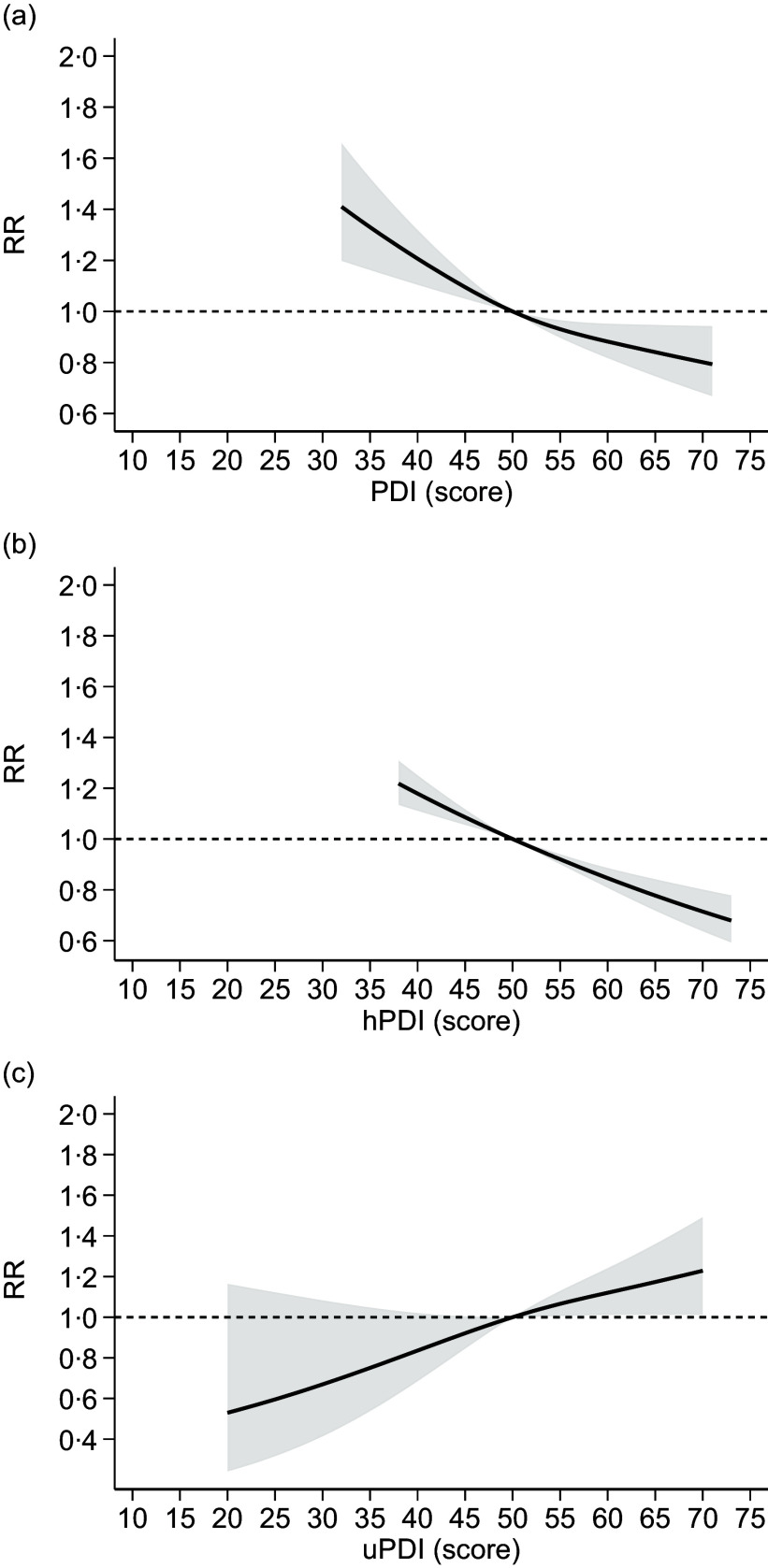
Dose–response meta-analysis of risk of type 2 diabetes according to adherence to plant-based dietary patterns ((a): PDI: plant-based diet index; (b): hPDI: healthy PDI; (c): uPDI: unhealthy PDI). Spline curve (solid line) with 95 % confidence limits (grey area). RR: risk ratio. The curves are designed using restricted cubic spline method using three knots at fixed cutpoints (tenth, fiftieth and ninetieth percentiles) and considering the median value (fiftieth) of such distribution as reference point. The short-dashed line represents the null effect, RR = 1.

In the dose–response meta-analysis, we were able to include results from fourteen studies, assessing adherence to plant-based indices reporting scores for each category of exposure, with higher scores indicating greater adherence. Overall, the analysis based on the assessment of overall PDI available in thirteen studies^(49,52,56,57,60,62,64,65,69,70,79)^ showed an inverse and almost linear relationship with T2D risk (Figure 4). Similarly, the analysis assessing the hPDI, based on eleven studies^(49,52,57,62,64,65,69,70,72)^, indicated a substantially linear, negative association with T2D risk for increasing adherence to hPDI (Figure 4). Conversely, the meta-analysis of results of the seven studies^(49,57,62,64,65,69,72)^ measuring adherence to uPDI showed a linear positive association with T2D risk for increasing levels of uPDI adherence (Figure 4).

Stratified analyses by region showed substantially similar results in Western populations to those obtained from the overall analysis, when the highest *v*. the lowest adherence to vegetarian dietary patterns were compared. In Asian populations, only adherence to lacto-ovo-vegetarian diet showed a negative association with T2D risk, although only one study for each of the other dietary patterns (i.e. vegan, pesco-vegetarian and semi-vegetarian) was available for the analysis (online Supplementary Figures S1–S2). Stratified analysis by region considering the highest *v*. the lowest adherence to plant-based dietary patterns showed similar results in both Western and Asian populations, with lower T2D risk for PDI and hPDI, and higher risk of uPDI, although the number of studies carried out in Asia is more limited (online Supplementary Figures S3–S4).

Similarly, a negative linear association between increasing PDI and hPDI adherence and T2D risk emerged both in Western and in Asian populations, although levels of adherence were generally higher in the former. Consistent with the forest-plot analysis, uPDI showed a positive association with T2D risk in Western populations, while for Asia, two studies were available and the analysis could not be performed (online Supplementary Figure S5).

### Sensitivity analyses

The analysis limited to cohort studies confirmed the overall RR comparing the highest *v*. the lowest adherence to vegetarian dietary patterns (online Supplementary Figure S6) as well as using plant-based dietary patterns (PDI, hPDI and uPDI), in both the forest plot (online Supplementary Figure S7) and in the dose–response meta-analysis (online Supplementary Figure S8).

Further restricting the analysis to cohort studies with more than 10 years of follow-up, we found a negative association between lacto-ovo-vegetarian (two studies) and semi-vegetarian diets (one study) and T2D risk, while one study for vegan diet showed null association (online Supplementary Figure S9). No studies were available for pesco-vegetarian diets with more than 10 years of follow-up. As regards plant-based patterns, results were almost identical to those in the main analysis in both the forest plot (online Supplementary Figure S10) and in the dose–response meta-analysis (online Supplementary Figure S11), although based on a lower number of studies.

Exclusion of the fifteen studies deemed at high risk of bias could allow an analysis only for lacto-ovo-vegetarian diet, showing a negative association with T2D risk, as no studies remained available for pesco-vegetarian and semi-vegetarian diets, and the one study on vegan diet showed null association (online Supplementary Figure S12). Finally, studies that assessed adherence using PDI, hPDI and uPDI showed almost identical results to those in the overall analysis, in both the forest plot (online Supplementary Figure S13) and in the dose–response meta-analysis (online Supplementary Figure S14), since no studies were judged at high risk of bias.

Funnel plots based on the different exposure assessment methods did not indicate risk of small-study effects, as also shown by results of Egger’s test (online Supplementary Figure S15), with no studies added in the trim-and-fill analysis. Finally, study-specific curves showed substantial homogeneous trends for all studies included in dose–response analyses except for one study in the overall PDI, showing a much steeper decrease in risk (online Supplementary Figure S16).

## Discussion

In this review, we found fairly consistent evidence that adherence to vegetarian and plant-based diets is associated with a lower risk of T2D. This was particularly true for studies comparing lacto-ovo-vegetarian compared with pesco- and semi-vegetarian diets. Similarly, we observed an inverse and almost linear association between overall PDI and disease risk, driven by increasing adherence to a hPDI, characterised by higher intakes of whole grains, fruits and vegetables. Conversely, our analyses showed that adherence to a uPDI may, in fact, increase T2D risk, which is likely due to a higher intake of processed foods and refined carbohydrates, although of plant-based origin.

Our review took advantage of newly available studies that contributed to the implementation of several stratified and sensitivity analyses compared with previous investigations on the same topic^(13,31)^. In addition, the availability of advanced statistical techniques allowed us to implement dose–response meta-analysis also for uPDI, never reported previously. As a consequence, our findings allowed us to investigate the relationship between vegetarian/vegan and plant-based diets in more depth. The important conclusion of this analysis was that their beneficial effects are only observed if accompanied by an increased intake of a range of ‘healthy’ foods, including whole grains, raw and cooked vegetables, legumes, nuts, fruits, vegetable oils and tea and coffee, not just by limiting the intake of meat products.

Mechanisms for the beneficial effects of increasing adherence to vegetarian and more broadly plant-based diets may include anti-inflammatory and antioxidant effects relating to the increased intake of fibre and polyphenols^(80–82)^ contained in whole grains, fruit and vegetables. These dietary factors are recognised to be associated with a lower risk of T2D in previous epidemiological studies^(23,83–86)^. Polyphenols have been shown to improve endothelial function, which has been correlated with insulin resistance and interactions with molecular targets that affect insulin signalling, resulting in an improvement in glycaemia and suppression of gluconeogenesis^(87,88)^. Insoluble fibre lowers post-prandial blood glucose and insulin through slower absorption and a reduced level of absorbed carbohydrate^(89)^. Fibre intake is associated with favourable profiles of gut microbiota^(90)^ that have been linked to decreased risk of T2D^(91,92)^. Similarly, adherence to plant-based and vegetarian diets can alter levels of prebiotics, microbial composition and production of microbial metabolites compared with omnivorous diet, thus affecting glycaemic control and T2D risk^(93–96)^. An additional mechanism of plant-based diets in lowering T2D risk is through weight control: in relation to this, experimental trials reported that the consumption of a vegetarian diet led to a reduction in body weight compared with an omnivorous diet^(97)^.

Subjects following vegetarian or plant-based diets demonstrated lower risk of insulin resistance^(53,98)^. Similarly, high intakes of saturated fatty acids are thought to decrease insulin sensitivity^(99,100)^, further supporting the detrimental effects of too high intake of meat products^(100,101)^. Interestingly, a study comparing the prevalence of impaired glucose tolerance amongst Australian women^(102)^ adhering to different diets (vegan, lacto-ovo vegetarian, pesco-vegetarian, semi-vegetarian and meat eaters) found lower impaired glucose tolerance in subjects following a vegetarian diet (0–1·2 %) compared with regular meat eaters (9·1 %). Source of proteins can also affect risk of T2D, with increased risk for moderate-high animal protein intake, whereas it is the opposite with higher intake of plant proteins^(103,104)^. Similarly, the switch in energy intake from animal to plant proteins was associated with lower T2D risk^(105)^. Finally, there are many studies that report a beneficial relationship between plant-based diets and other chronic diseases, including metabolic syndrome and dyslipidaemia^(106–108)^.

In spite of the recognised beneficial effects of vegetarian and vegan diets, several studies and guidelines indicate that animal foods might not be totally excluded due to their important contribution to the dietary intake of certain nutrients, including *n*-3 fatty acids, calcium and vitamin D, vitamin B_12_, selenium, zinc and iron^(109–113)^. There is a need to properly plan food intake in order to ensure adequate intake of these nutrients^(114)^ and/or to use supplements and fortified food products^(115)^. It should be noted that in the included studies investigating adherence to PDI, subjects in the highest category reported limited but not zero intake of animal products. As also indicated by the differential (and even opposite) results for hPDI and uPDI in the relationship with T2D risk, limitation of intake of animal foods alone does not appear to be enough for disease prevention^(116)^. Recommendations to limit processed foods like refined grains, sweets and sugar-sweetened beverages, along with weight control and increased physical activity, and lower intake of other dietary and environmental factors associated with increased T2D risk^(117–120)^ are clearly fundamental^(26–28,30)^. Therefore, our findings suggest that guidelines for T2D prevention should consider the inclusion of a higher adherence to healthy plant-based and vegetarian diets. These dietary recommendations linked to our findings are in line with the beneficial role of other dietary patterns considered healthy in lowering T2D risk, especially the Mediterranean diet and the Dietary Approach to Stop-Hypertension^(121,122)^ characterised by lower intake of salt, highly processed foods and sweet products in addition to meat and meat products^(123,124)^. Nonetheless, beneficial effects of vegan/vegetarian diet have also been reported for the management of T2D, lowering Hb1Ac levels and improving insulin sensitivity^(125,126)^, especially if characterised by high-protein content^(127)^.

Some limitations of the review should be noted. The restricted number of studies on some subtypes of vegetarian diets hampered the implementation of meaningful stratified analyses, especially those of cohort design with longer follow-up. In addition, the lack of reporting of quantitative assessment of adherence to vegan and vegetarian diets prevented a dose–response analysis of such associations. In addition, there was some heterogeneity in outcome ascertainment (T2D diagnosis) across the studies, being generally self-reported in studies investigating vegetarian and vegan diets, while relying on medical records when using plant-based indices. Nonetheless, we took into account these methodological issues within the risk of bias assessment, and we found substantially homogenous results among included studies in both forest-plots and dose–response curves, as when we excluded studies at overall high risk of bias. Specifically, none of the studies using plant-based indices for exposure assessment were judged at high risk of bias. Strengths of the review include the implementation of analysis stratified by type of vegan/vegetarian diet as well as for overall PDI and divided into hPDI and uPDI. Finally, low risk of publication bias further strengthens the quality of our findings, especially when considering cohort studies only, characterised by higher methodological quality compared with the remaining observational studies.

In conclusion, this review indicates that healthy plant foods, including whole grains, vegetables, fruits, legumes and nuts, may protect against T2D in an almost linear fashion, while unhealthy plant-based diets are not beneficial and may even increase disease risk at high levels.

## Supporting information

Murciano et al. supplementary material Murciano et al. supplementary material

## Supplementary material

For supplementary material/s referred to in this article, please visit https://doi.org/10.1017/S0007114525104157
