# Supplementary material for: Plant-based diets and risk of type 2 diabetes: systematic review and dose–response meta-analysis
Source: Br J Nutr. 2025 Aug 12;134(4):277–96. doi: 10.1017/S0007114525104157 (PMC12530962; doi:10.1017/S0007114525104157)
Supplement: Murciano et al. supplementary material [file S0007114525104157sup001.docx]

**sSUPPLEMENTARY MATERIAL**

**Supplementary Table S1**. Details of literature search on online databases.

| **Database** | **Search string** |
| --- | --- |
| PubMed/MEDLINE | ("Diet, Vegetarian"[Mesh] OR vegetarian diet[tiab] OR lacto-vegetarian diet[tiab] OR plant based diet[tiab] OR Plant Based Nutrition[tiab] OR Lacto Ovo Vegetarian Diet[tiab] OR Vegetarianism[tiab] OR "Diet, Vegan"[MH] OR Vegan diet[tiab]) OR Veganism[tiab] AND (Diabetes[tiab] OR diabetes[MH]) |
| EMBASE | 'diabetes mellitus'/exp AND ('vegetarian diet' OR 'lactovegetarian diet' OR 'lactoovovegetarian diet' OR vegetarianism OR 'vegan diet') |

**Supplementary Table S2**. Criteria adopted for risk of bias assessment using Risk of Bias for in Non-randomized Studies of Exposures (ROBINS-E) tool.

| **Domains** | **Criteria** |
| --- | --- |
| Bias due to confounding | Studies are considered at **moderate** risk of bias if they considered age in the confounding factors. Studies are considered at **low** risk of bias if they considered also BMI in the adjustment factors. Studies are considered at **high** risk of bias if adjusting factors are not reported. |
| Bias in selecting participants in the study | Studies are considered at **low** risk of bias if selection of eligible participants was not linked to a predominantly plant-based diet. Studies are considered at **moderate** risk of bias if participant selection was linked to a predominantly plant-based diet. Studies are considered at **high** risk of bias if information of modality of selection of participants is missing. |
| Bias in exposure classification | Studies are considered at **low** risk of bias if exposure classification was performed using a validated questionnaire. Studies are considered at **moderate** risk of bias if questionnaire validation was not specified. Studies are considered at **high** risk of bias if they relied on self-report for exposure classification or criteria are not reported. |
| Bias in departure from intended exposure | Studies are considered at **low** risk of bias if exposure doses were reported, while at **moderate** risk of bias if exposure levels were reported but not the doses in each category. Studies are considered at **high** risk of bias if exposure levels are not reported. |
| Bias due to missing data | Studies are considered at **low** risk of bias if less than 10% of participants were excluded to missing data, while at **moderate** risk of bias if less than 20%. Studies with higher proportion (≥20%) are considered at **high** risk of bias. |
| Bias in outcome measurement | Studies are considered at **low** risk of bias if outcome assessment was based on access to disease registry or use of international guideline for definition of diabetic status. Studies are considered at **moderate** risk of bias if outcome assessment was based on self-report but with subsequent external validation. Studies are considered at **high** risk of bias if outcome assessment was based on self-report only without external validation, or was not based on international guidelines, or if information about outcome assessment was missing. |
| Bias in selection of reported results | Studies are considered at **low** risk of bias if they reported a prior publication of the protocol or data are made available in a public and accessible repository. Study are considered at **moderate** risk of bias if they presented outcome measures and analyses consistent with a priori plan outlined in the manuscript. Studies are considered at **high** risk of bias if no protocol was available and the a priori plan was not outlined |
| Overall risk of bias | If at least one domain was found at **high** risk of bias, the overall risk was considered **high**. If at least one domain was found at **moderate** risk of bias, the overall risk was considered **moderate**. If all domains were at **low** risk of bias, the overall risk was considered **low**. |

**Supplementary Table S3.** Risk of bias assessment using Risk of Bias for in Non-randomized Studies of Exposures (ROBINS-E) tool.

| **Reference** | **Bias due to confounding** | **Bias in selecting participants in the study** | **Bias in exposure classification** | **Bias in departure from intended exposure** | **Bias due to missing data** | **Bias in outcome measurement** | **Bias in selection of reported results** | **Study-level RoB Judgment** |
| --- | --- | --- | --- | --- | --- | --- | --- | --- |
| *Cohort* |  |  |  |  |  |  |  |  |
| Bhupathiraju *et al.* ^(49)^ | Low | Low | Low | Low | Moderate | Low | Low | Low |
| Boonpor *et al.* ^(50)^ | Low | Low | Low | Moderate | Low | Low | Low | Low |
| Chen *et al.* ^(52)^ | Low | Low | Low | Low | Moderate | Moderate | Low | Moderate |
| Chen *et al.* ^(53)^ | Low | Low | Low | Low | Low | Low | Low | Low |
| Chiu *et al.* ^(55)^ | Low | Moderate | High | High | Low | Low | Low | High |
| Choi *et al.* ^(56)^ | Low | Low | Low | Low | Low | Low | Low | Low |
| Flores *et al.* ^(57)^ | Low | Low | Low | Low | Low | Low | Low | Low |
| Kim and Giovannucci ^(62)^ | Low | Low | Low | Low | Low | Low | Low | Low |
| Koloverou *et al.* ^(63)^ | Low | Low | Low | Moderate | High | Low | Low | High |
| Laouali *et al.* ^(64)^ | Low | Low | Low | Low | Low | Moderate | Low | Low |
| Lv *et al.* ^(65)^ | Low | Low | Low | Low | Low | Low | Low | Low |
| Papier *et al.* ^(67)^ | Low | Low | Low | Moderate | Low | Low | Low | Low |
| Satija *et al.* ^(70)^ | Low | Low | Low | Low | Moderate | Moderate | Low | Moderate |
| Sullivan *et al.* ^(69)^ | Low | Low | Low | Low | Low | Low | Low | Low |
| Thompson *et al.* ^(72)^ | Low | Low | Low | Low | Low | Low | Low | Low |
| Tonstad *et al.* ^(73)^ | Low | Moderate | Low | Moderate | Moderate | Moderate | Low | High |
| Vang *et al.* ^(75)^ | Low | Moderate | Low | Moderate | Moderate | High | Low | High |
| *Cross-sectional* |  |  |  |  |  |  |  |  |
| Agrawal *et al.* ^(47)^ | Low | Low | Moderate | Moderate | Low | High | Low | High |
| Bharati *et al.* ^(48)^ | Low | Low | Moderate | Moderate | Moderate | Low | Low | Moderate |
| Brathwaite *et al.* ^(51)^ | High | Moderate | Moderate | High | Moderate | Low | Low | High |
| Chiu *et al.* ^(54)^ | Low | Moderate | Low | Moderate | Low | Low | Low | Moderate |
| Fraser ^(58)^ | Moderate | Moderate | Low | Moderate | Moderate | Moderate | Low | High |
| Golebiowska *et al.* ^(59)^ | Low | Low | High | High | Low | High | Low | High |
| Heidarzadeh-Esfahani *et al.* ^(60)^ | Moderate | Low | Low | Low | Low | Low | Low | Low |
| Jaacks *et al.* ^(61)^ | Moderate | Low | Moderate | Moderate | Moderate | Low | Low | High |
| Misra *et al.* ^(66)^ | Moderate | Low | High | High | Low | High | Low | High |
| Ponzio *et al.* ^(68)^ | Low | Low | High | High | Low | High | Low | High |
| Shridhar *et al.* ^(71)^ | High | Low | Low | Moderate | Low | Low | Low | High |
| Tonstad *et al.* ^(74)^ | Low | Moderate | Low | Moderate | High | Moderate | Low | High |
| Yang *et al.* ^(76)^ | Low | Low | Low | Moderate | Low | Low | Low | Low |
| Yogal *et al.* ^(77)^ | Moderate | Low | High | High | Low | Low | Low | High |
| Zhang *et al.* ^(78)^ | Moderate | Low | Moderate | Moderate | Low | Low | Low | Moderate |
| Zhang *et al.* ^(79)^ | Low | Low | Low | Low | Low | Low | Low | Low |

**Supplementary Figure S1**. Forest plot of the included studies regarding risk of type 2 diabetes comparing the highest versus the lowest adherence to different plant-based dietary patterns in Western populations (VEGAN: vegan diet; LOV: lacto-ovo-vegetarian diet; PV: pesco-vegetarian diet; SV: semi-vegetarian diet). RR: risk ratio. CI: confidence interval. The area of each grey square is proportional to the inverse of the variant of the estimated RR, and horizontal lines represent the 95% CI. Black diamonds represent point estimates of overall RR for each group. The solid vertical line represents null effect (RR=1).

**Supplementary Figure S2**. Forest plot of the included studies regarding risk of type 2 diabetes comparing the highest versus the lowest adherence to different plant-based dietary patterns in Asian populations (VEGAN: vegan diet; LOV: lacto-ovo-vegetarian diet; PV: pesco-vegetarian diet; SV: semi-vegetarian diet). RR: risk ratio. CI: confidence interval. The area of each grey square is proportional to the inverse of the variant of the estimated RR, and horizontal lines represent the 95% CI. Black diamonds represent point estimates of overall RR for each group. The solid vertical line represents null effect (RR=1).

**Supplementary Figure S3**. Forest plot of the included studies regarding risk of type 2 diabetes comparing the highest versus the lowest adherence to different plant-based dietary patterns in Western populations (PDI: plant-based diet index; hPDI: healthy PDI; uPDI: unhealthy PDI) RR: risk ratio. CI: confidence interval. The area of each grey square is proportional to the inverse of the variant of the estimated RR, and horizontal lines represent the 95% CI. Black diamonds represent point estimates of overall RR for each group. The solid vertical line represents null effect (RR=1).

**Supplementary Figure S4**. Forest plot of the included studies regarding risk of type 2 diabetes comparing the highest versus the lowest adherence to different plant-based dietary patterns in Asian populations (PDI: plant-based diet index; hPDI: healthy PDI; uPDI: unhealthy PDI) RR: risk ratio. CI: confidence interval. The area of each grey square is proportional to the inverse of the variant of the estimated RR, and horizontal lines represent the 95% CI. Black diamonds represent point estimates of overall RR for each group. The solid vertical line represents null effect (RR=1).

**Supplementary Figure S5.** Dose-response meta-analysis of risk of type 2 diabetes according adherence to plant-based dietary patterns (A: PDI: plant-based diet index; B: hPDI: healthy PDI; C: uPDI: unhealthy PDI in Western populations; D: PDI: plant-based diet index and E: hPDI: healthy PDI in Asian populations) Spline curve (solid line) with 95% confidence limits (grey area). RR: risk ratio. The curves are designed using restricted cubic spline method using 3 knots at fixed cutpoints (10th, 50th and 90th percentiles) and considering the median value (50th) of such distribution as reference point. The short-dashed line represents the null effect, RR=1.

**
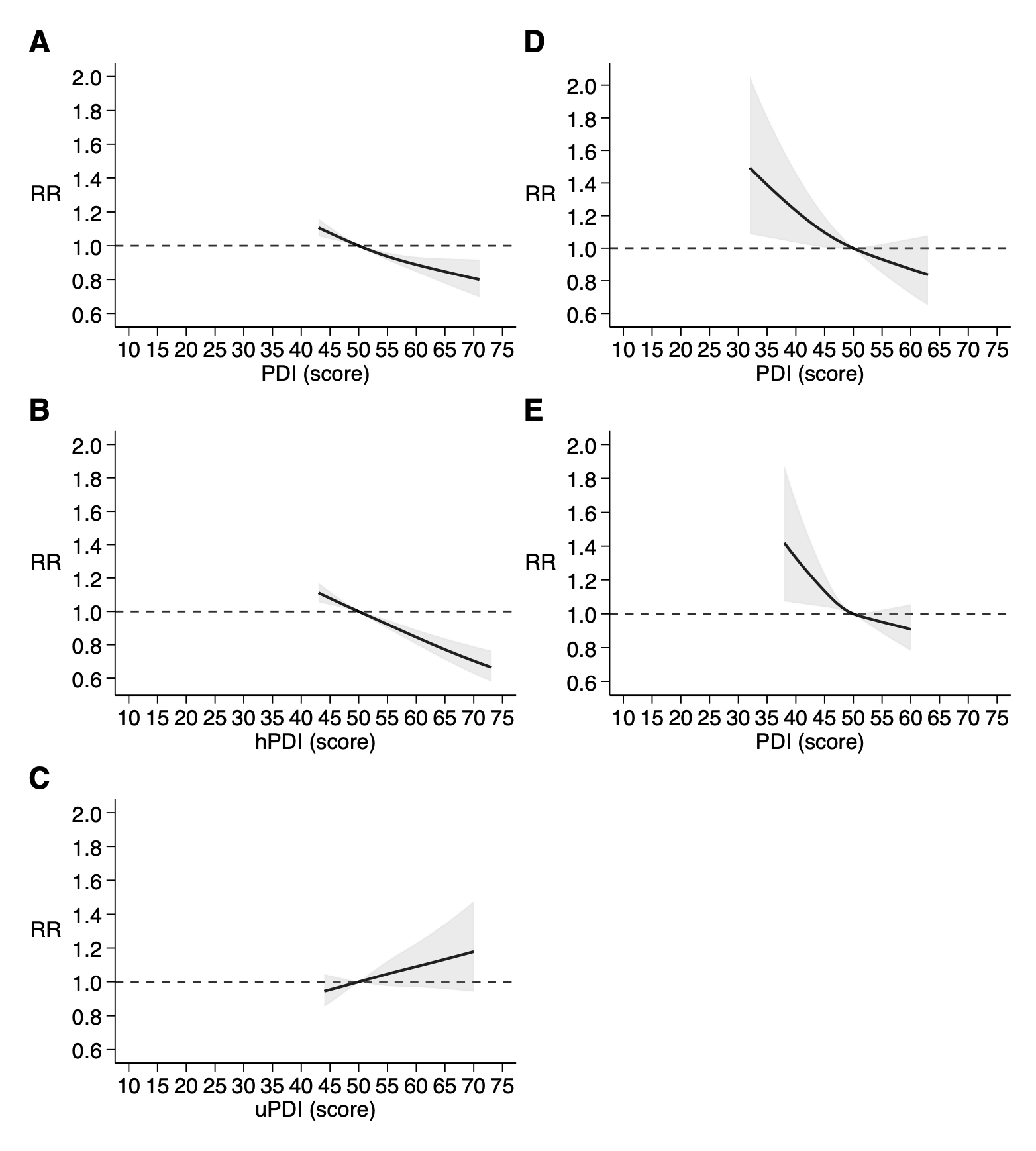
**

**Supplementary Figure S6**. Forest plot of the included studies regarding risk of type 2 diabetes comparing the highest versus the lowest adherence to different plant-based dietary patterns in cohort studies only (VEGAN: vegan diet; LOV: lacto-ovo-vegetarian diet; PV: pesco-vegetarian diet; SV: semi-vegetarian diet). RR: risk ratio. CI: confidence interval. The area of each grey square is proportional to the inverse of the variant of the estimated RR, and horizontal lines represent the 95% CI. Black diamonds represent point estimates of overall RR for each group. The solid vertical line represents null effect (RR=1).

**Supplementary Figure S7**. Forest plot of the included studies regarding risk of type 2 diabetes comparing the highest versus the lowest adherence to different plant-based dietary patterns in cohort studies only (PDI: plant-based diet index; hPDI: healthy PDI; uPDI: unhealthy PDI) RR: risk ratio. CI: confidence interval. The area of each grey square is proportional to the inverse of the variant of the estimated RR, and horizontal lines represent the 95% CI. Black diamonds represent point estimates of overall RR for each group. The solid vertical line represents null effect (RR=1).

**Supplementary Figure S8.** Dose-response meta-analysis of risk of type 2 diabetes according to adherence to plant-based dietary patterns in cohort studies only (A: PDI: plant-based diet index; B: hPDI: healthy PDI; C: uPDI: unhealthy PDI). Spline curve (solid line) with 95% confidence limits (grey area). RR: risk ratio. The curves are designed using restricted cubic spline method using 3 knots at fixed cutpoints (10th, 50th and 90th percentiles) and considering the median value (50th) of such distribution as reference point. The short-dashed line represents the null effect, RR=1.


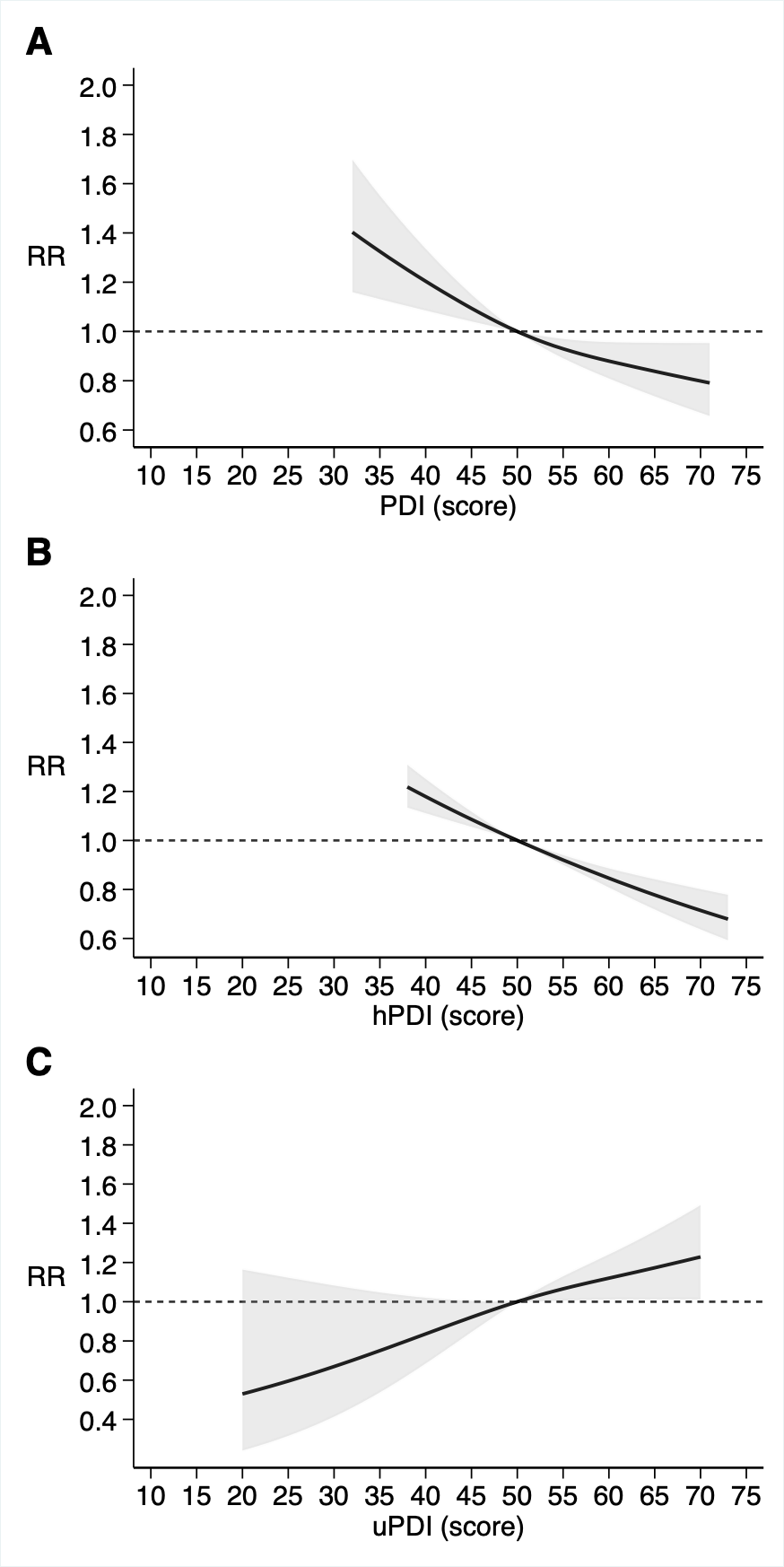


**Supplementary Figure S9**. Forest plot of the included studies regarding risk of type 2 diabetes comparing the highest versus the lowest adherence to different plant-based dietary patterns in cohort studies only with follow-up ≥10 years (VEGAN: vegan diet; LOV: lacto-ovo-vegetarian diet; PV: pesco-vegetarian diet; SV: semi-vegetarian diet). RR: risk ratio. CI: confidence interval. The area of each grey square is proportional to the inverse of the variant of the estimated RR, and horizontal lines represent the 95% CI. Black diamonds represent point estimates of overall RR for each group. The solid vertical line represents null effect (RR=1).

**Supplementary Figure S10**. Forest plot of the included studies regarding risk of type 2 diabetes comparing the highest versus the lowest adherence to different plant-based dietary patterns in cohort studies only with follow-up ≥10 years (PDI: plant-based diet index; hPDI: healthy PDI; uPDI: unhealthy PDI) RR: risk ratio. CI: confidence interval. The area of each grey square is proportional to the inverse of the variant of the estimated RR, and horizontal lines represent the 95% CI. Black diamonds represent point estimates of overall RR for each group. The solid vertical line represents null effect (RR=1).

**Supplementary** **Figure S11.** Dose-response meta-analysis of risk of type 2 diabetes according to adherence to plant-based dietary patterns in cohort studies only with follow-up ≥10 years (A: PDI: plant-based diet index; B: hPDI: healthy PDI; C: uPDI: unhealthy PDI). Spline curve (solid line) with 95% confidence limits (grey area). RR: risk ratio. The curves are designed using restricted cubic spline method using 3 knots at fixed cutpoints (10th, 50th and 90th percentiles) and considering the median value (50th) of such distribution as reference point. The short-dashed line represents the null effect, RR=1.


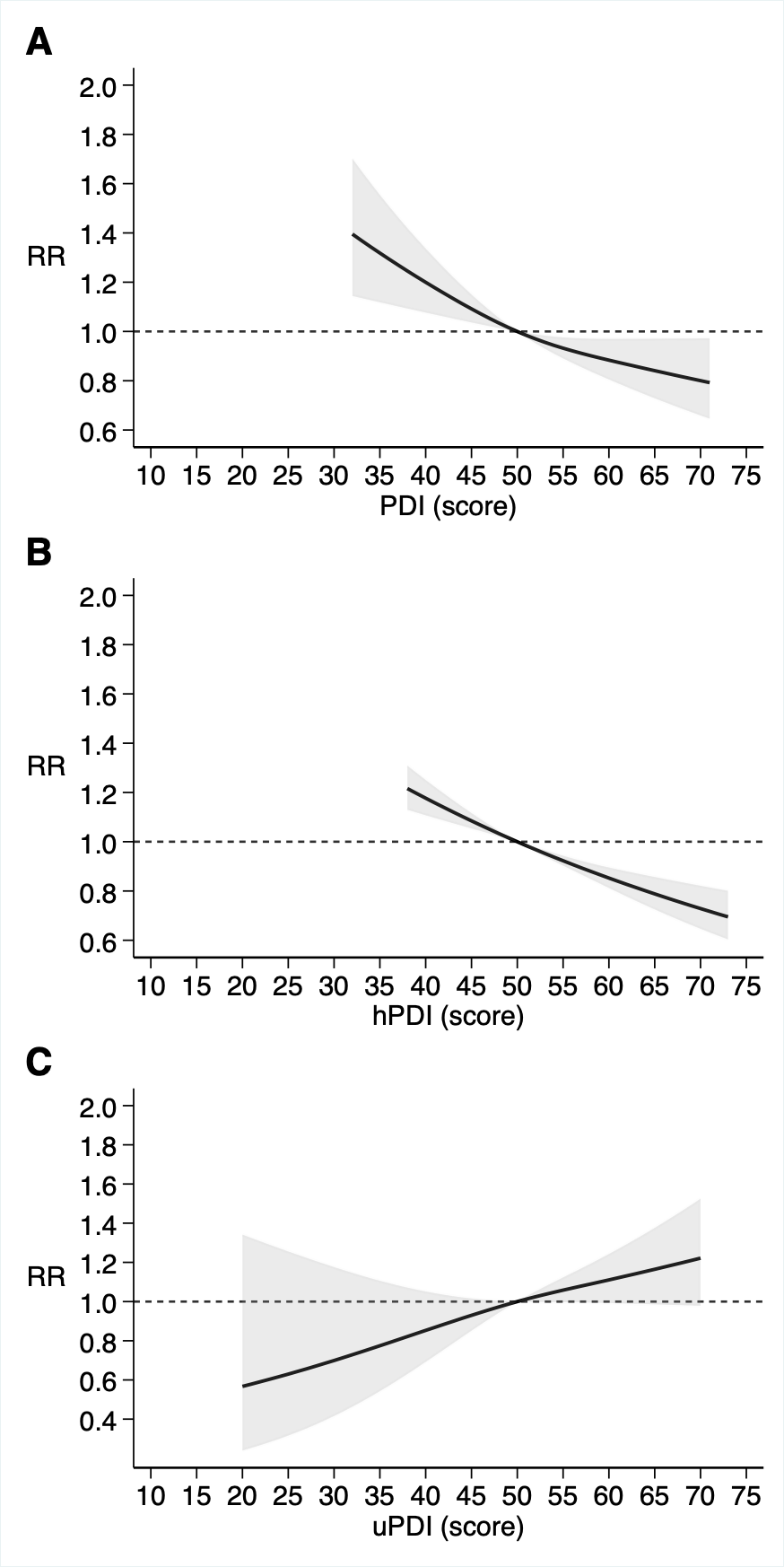


**Supplementary** **Figure S12**. Forest plot of the included studies regarding risk of type 2 diabetes comparing the highest versus the lowest adherence to different plant-based dietary patterns excluding studies at high risk of bias (VEGAN: vegan diet; LOV: lacto-ovo-vegetarian diet). RR: risk ratio. CI: confidence interval. The area of each grey square is proportional to the inverse of the variant of the estimated RR, and horizontal lines represent the 95% CI. Black diamonds represent point estimates of overall RR for each group. The solid vertical line represents null effect (RR=1).

**Supplementary** **Figure S13**.Forest plot of the included studies regarding risk of type 2 diabetes comparing the highest versus the lowest adherence to different plant-based dietary patterns excluding studies at high risk of bias (PDI: plant-based diet index; hPDI: healthy PDI; uPDI: unhealthy PDI) RR: risk ratio. CI: confidence interval. The area of each grey square is proportional to the inverse of the variant of the estimated RR, and horizontal lines represent the 95% CI. Black diamonds represent point estimates of overall RR for each group. The solid vertical line represents null effect (RR=1).

**Supplementary** **Figure S14.** Dose-response meta-analysis of risk of type 2 diabetes according to adherence to plant-based dietary patterns excluding studies at high risk of bias (A: PDI: plant-based diet index; B: hPDI: healthy PDI; C: uPDI: unhealthy PDI). Spline curve (solid line) with 95% confidence limits (grey area). RR: risk ratio. The curves are designed using restricted cubic spline method using 3 knots at fixed cutpoints (10th, 50th and 90th percentiles) and considering the median value (50th) of such distribution as reference point. The short-dashed line represents the null effect, RR=1.


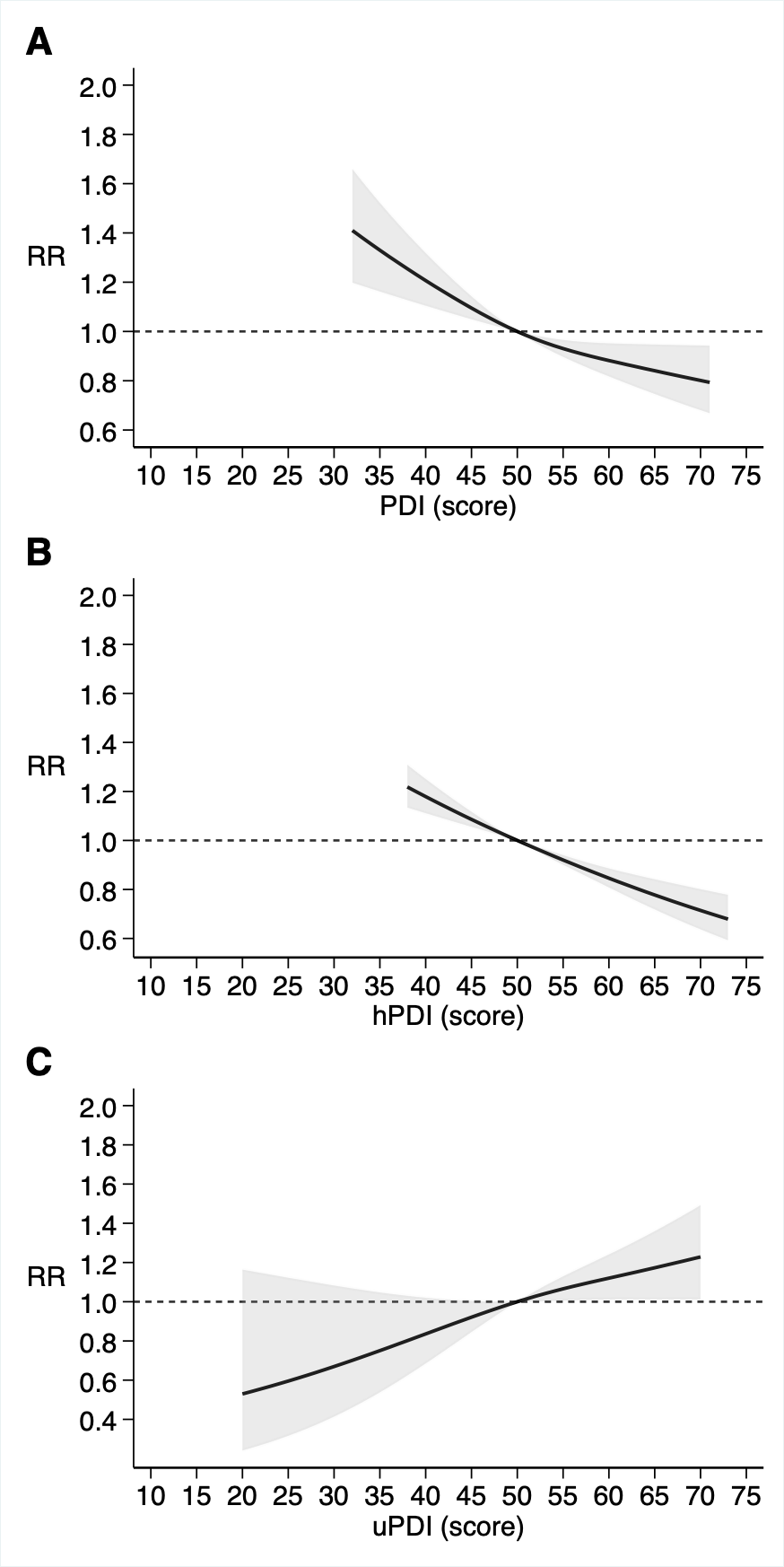


**Supplementary Figure S15.** Funnel plots for publication bias (VEGAN: vegan diet; LOV: lacto-ovo-vegetarian diet; PV: pesco-vegetarian diet; SV: semi-vegetarian diet; PDI: plant-based diet index; hPDI: healthy PDI; uPDI: unhealthy PDI).

**Supplementary Figure S16.** Dose-response meta-analysis of risk of type 2 diabetes according to adherence to plant-based dietary patterns excluding studies at high risk of bias (A: PDI: plant-based diet index; B: hPDI: healthy PDI; C: uPDI: unhealthy PDI). Spline curve (solid line) with 95% confidence limits (grey area) and the study-specific trends showing the influence of variation across studies (grey solid lines) RR: risk ratio. The curves are designed using restricted cubic spline method using 3 knots at fixed cutpoints (10th, 50th and 90th percentiles) and considering the median value (50th) of such distribution as reference point. The short-dashed line represents the null effect, RR=1.

**
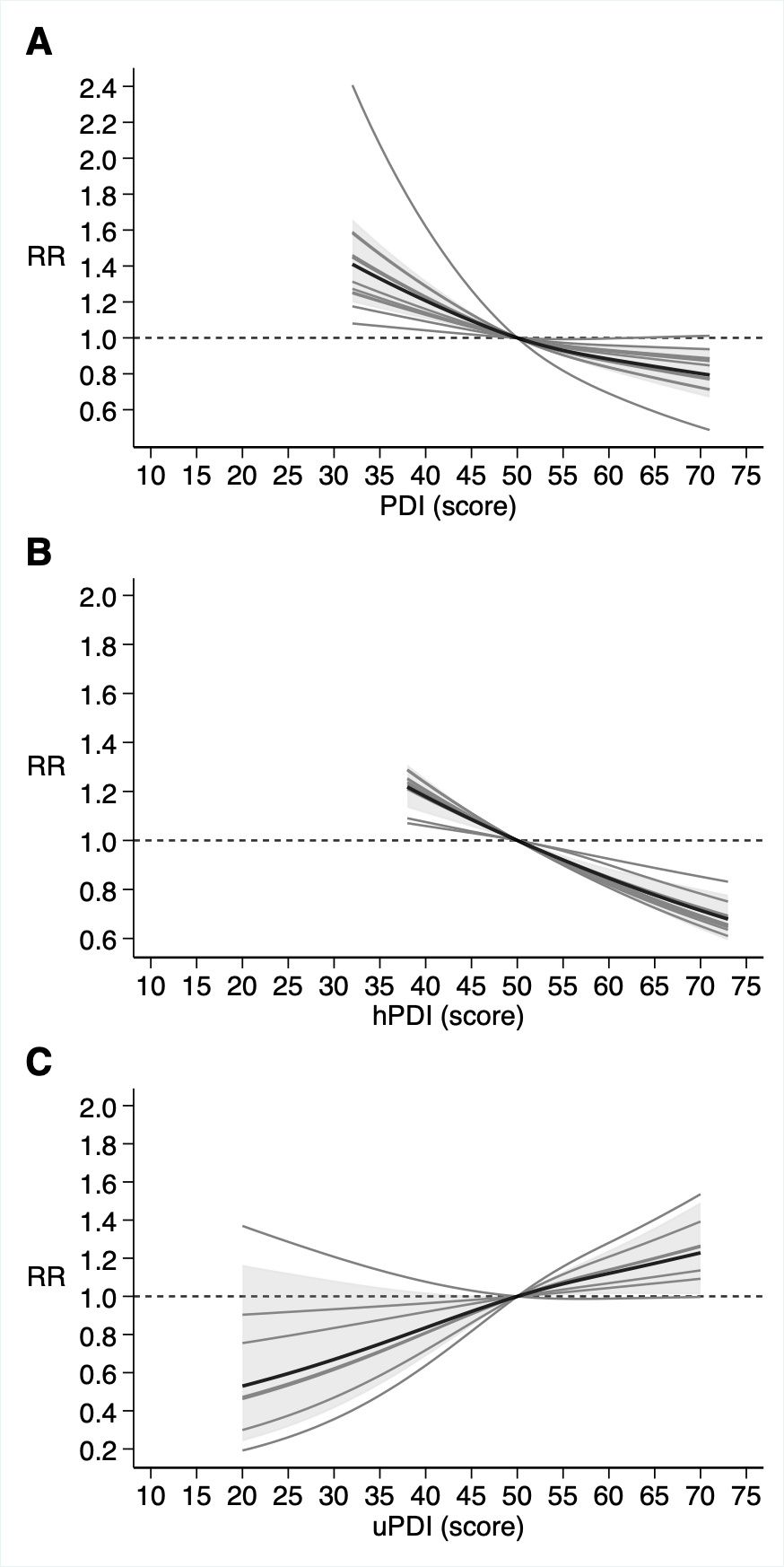
**
